# Effects of Arsenic and Mercury Exposure on Thyroid Dysfunction: An Evidence-Based Risk Assessment Using the TRAEC Strategy

**DOI:** 10.3390/toxics14090795

**Published:** 2026-09-08

**Authors:** Junyi Lv, Hanjun Chen, Mohammed Alqudaimi, Ziye Xia, Weining Li, Chao Dong, Rigoberto Flores Carpintero, Yankai Xia, Yu Xia, Qingfeng Chen

**Affiliations:** 1 Key Laboratory of Modern Toxicology of Ministry of Education, School of Public Health, Nanjing Medical University, Nanjing 211166, China; 2025110466@stu.njmu.edu.cn (J.L.); 15212454202@163.com (H.C.); m.alqudaimi4@gmail.com (M.A.); xiayy@stu.njmu.edu.cn (Z.X.); winnielee1206@gmail.com (W.L.); terencedong@njmu.edu.cn (C.D.); floresc_rigoberto@outlook.com (R.F.C.); yankaixia@njmu.edu.cn (Y.X.); 2 State Key Laboratory of Reproductive Medicine and Offspring Health, Center for Global Health, School of Public Health, Nanjing Medical University, Nanjing 211166, China; 3 Suzhou Center for Disease Control and Prevention, Suzhou 215131, China; 4 Nanchang Medical College, Nanchang 330052, China

**Keywords:** arsenic, mercury, thyroid, risk assessment, TRAEC strategy

## Abstract

Thyroid homeostasis is essential for human health, and arsenic (As) and mercury (Hg) have drawn considerable attention due to their bioaccumulation and potential health risks. However, evidence on their thyroid-disrupting effects remains inconsistent and largely derived from cross-sectional studies, hindering causal inference and risk integration. Here, a meta-analysis was first conducted, which confirmed that both As (Cohen’s d = 0.45, 95% CI: 0.10, 0.81) and Hg (Cohen’s d = 0.40, 95% CI: 0.16, 0.65) exposure were associated with an increased risk of thyroid dysfunction. To further clarify temporality, we analyzed prospective cohort data from 800 adult women in Jiangsu, China. Results showed that As exposure was linearly and positively associated with free thyroxine (FT4) (β = 0.19, 95% CI: 0.01, 0.37), while Hg showed an inverted U-shaped dose–response relationship with FT4 levels. Based on the above epidemiological evidence, we further integrated in vivo and in vitro evidence from published related literature, applied the Targeted Risk Assessment for Environmental Chemicals (TRAEC, ver.1.1) strategy, and obtained final scores of 7.79 for As and 7.09 for Hg (out of 10), indicating a moderate level of thyroid-related health risk. Collectively, these findings suggest that As and Hg exposure may disturb thyroid function, supporting the need for targeted mitigation and further mechanistic research.

## 1. Introduction

The thyroid gland plays a critical role in human metabolism, growth and development, and its homeostasis is finely regulated by the hypothalamic-pituitary-thyroid (HPT) axis [1,2,3]. This regulatory system is highly vulnerable to environmental chemicals, and exposure to toxic metal(loid)s is widely recognized as a major risk factor for thyroid dysfunction [4]. Compared with accumulation in other tissues, toxic metal(loid)s may accumulate more readily in the thyroid gland, thereby interfering with thyroid hormone synthesis, secretion, transport, and metabolism, ultimately disrupting endocrine homeostasis [5,6].

Among these toxic metal(loid)s, arsenic (As) and mercury (Hg), also priority environmental pollutants classified by both China and the United States [7], stand out as the most representative pollutants in thyroid health research [8]. They are widely distributed in soil, water bodies, and the food chain, primarily originating from anthropogenic sources such as industrial emissions, agricultural activities, and coal combustion [7,9]. In agricultural soils across China, Hg exhibited the highest bioaccumulation and ecological risk indices, while As showed the greatest non-carcinogenic hazard quotient among priority metal(loid)s in dietary exposure assessments [10,11]. Importantly, this non-carcinogenic hazard quotient does not account for the established carcinogenicity of arsenic, which is recognized as a human carcinogen. In China, As and Hg in some regions have been reported to account for 92.9–98.8% of the potential ecological risks posed by metal(loid)s. Surveys in some areas further confirmed that As and Hg were not only the dominant sources of ecological risk in local sediments but also exhibited significant bioaccumulation in the human body [12]. Moreover, epidemiological evidence suggests that exposure to As and Hg is closely associated with a spectrum of thyroid dysfunctions, including hypothyroidism, hyperthyroidism, autoimmune thyroid diseases, and thyroid cancer [13,14,15]. Collectively, their widespread environmental exposure and well-documented thyroid-disrupting effects justify focusing on these two metal(loid)s as key environmental contributors to thyroid dysfunction. It should be noted that not all regions face equally high exposure.

However, most existing epidemiological evidence derives from cross-sectional studies, which cannot clarify the temporal sequence between As and Hg exposure and alterations in thyroid hormone levels, thus limiting causal inference. Meanwhile, high heterogeneity across studies has led to inconsistent findings. Furthermore, the specific mechanisms by which As and Hg disrupt thyroid hormones balance remain incompletely understood. Together, current investigations remain fragmented and heterogeneous with insufficient robust causal evidence, hindering the integration of available data and a comprehensive assessment of thyroid health risks associated with As and Hg exposure.

To address these significant research gaps, we first conducted a meta-analysis to summarize the overall association of As and Hg exposure with thyroid dysfunction. Then, we utilized a prospective cohort in Jiangsu, China with both As and Hg in drinking water well below the national standard limits [16] to examine the associations between As and Hg exposure during the second trimester and subsequently assessed thyroid function during the third trimester. Furthermore, we applied the Targeted Risk Assessment for Environmental Chemicals (TRAEC, ver.1.1) strategy [17,18] to integrate epidemiological, in vivo, and in vitro evidence, enabling a standardized, transparent, and quantitative assessment of the related risks. Accordingly, this study aims to combine population-based cohort evidence with the structured TRAEC framework to comprehensively assess the thyroid-related health risks posed by As and Hg exposure.

## 2. Materials and Methods

### 2.1. Meta-Analysis

To assess the impact of As and Hg exposure on thyroid health, a systematic meta-analysis was performed. A systematic search of the PubMed and Web of Science databases was conducted to identify studies published up to 18 March 2026, investigating the association between As or Hg exposure and thyroid dysfunction. Study selection was done by using Preferred Reporting Items for Systematic Reviews and Meta-Analyses (PRISMA) guidelines. The detailed search strings for each database are provided in Supplemental Table S1. Subsequently, publication types such as meta-analyses, reviews, conference abstracts, case reports, and guidelines were excluded based on title and abstract screening. Finally, the remaining articles underwent full-text review, during which studies deemed irrelevant or lacking critical data were excluded.

The inclusion criteria were as follows: (a) cohort, cross-sectional, or case–control studies; (b) studies examining the effect of As or Hg exposure on thyroid abnormalities; and (c) epidemiological studies providing quantitative measures, such as risk ratios (RR), odds ratios (OR), or hazard ratios (HR) with corresponding 95% confidence intervals (95% CI). The exclusion criteria were as follows: (a) duplicate, non-original, or irrelevant studies, or those not meeting the above criteria; (b) studies with incomplete exposure or outcome data, or missing essential information; and (c) studies from which relevant data could not be extracted.

Study quality was assessed using the Newcastle-Ottawa Scale (NOS) and its adaptation for cross-sectional studies. To standardize effect size measures across the included epidemiological studies, various reported effect estimates were converted into a standardized mean difference (Cohen’s d) as the primary effect indicator [19]. Specifically, for OR/RR/HR, the conversion was based on the natural logarithm of the effect estimate. For regression coefficients (β), Cohen’s d was calculated using the SD of the exposure and outcome variables. For correlation coefficients (r), the conversion followed a standard transformation formula. Detailed procedures and formulas for effect-size conversion were based on the TRAEC 1.1 Scoring Guidance and are available on the TRAEC Web (https://traec.njmu.edu.cn/). Effect sizes were then pooled using a random-effects model [20]; results from a fixed-effects model are also presented for comparison. Heterogeneity was quantified with the I^2^ statistic [21]. Subgroup analyses were performed to explore the potential sources of heterogeneity, and publication bias was assessed using funnel plots and the Egger test.

### 2.2. Study Population

To address the inherent limitations of cross-sectional evidence summarized in the meta-analysis, a prospective cohort study was further conducted to assess the causal relationship between As/Hg exposure and thyroid dysfunction.

The study population consisted of adult women recruited at the Affiliated Maternity and Child Health Care Hospital of Nanjing Medical University between 2017 and 2018. Serum samples were collected and thyroid function was assessed during both the second-trimester (24–26 weeks of gestation) and third-trimester (28–32 weeks of gestation) follow-up visits. In the present study, As and Hg concentrations were measured in serum samples collected during the second trimester, and their associations with subsequent thyroid function during the third trimester were evaluated.

A total of 1527 adult women were initially enrolled. Participants were excluded according to the following criteria (Supplemental Figure S1): (i) use of medications affecting thyroid function within the past 3 months (*n* = 35); (ii) history of or current thyroid dysfunction, hyperlipidemia, or hypertension (*n* = 224); (iii) lack of serum trace element testing (*n* = 20); and (iv) lack of serum thyroid hormone level testing (*n* = 448). After exclusions, 800 adult women were included in the final analysis.

This study received ethical approval, with details provided in the Supplementary Materials. Written informed consent was obtained from all participants. All serum samples were collected using trace-element-free tubes and stored at −80 °C until analysis to prevent contamination and degradation.

### 2.3. Serum As and Hg Measurement

To ensure consistency in the testing conditions and comparability of the results, all serum samples were centrally analyzed at the Key Laboratory of Modern Toxicology, Ministry of Education, Nanjing Medical University, using the same instruments and standardized analytical procedures. Serum concentrations of As and Hg were measured as follows. Samples were digested with a diluent (0.05% [*v*/*v*] Triton X-100, 0.5% [*v*/*v*] nitric acid plus 10 mg/L internal standards including Sc, Y, In, Tb, and B) and analyzed using an iCAP Q series inductively coupled plasma mass spectrometer (ICP-MS; Thermo Fisher Scientific, Bremen, Germany).

The blank samples were prepared using the same diluent formulation without serum. To monitor analytical stability and accuracy, one standard quality control sample (Seronorm Trace Elements Serum L-1, ref. 203105, SERO AS, Billingstad, Norway) was analyzed after every 20 study samples, and a pooled quality control sample (prepared from 10% of the study samples) was run after every 10 samples. All analyses were completed within 17 days to minimize the inter-batch variation. The relative standard deviation (RSD) for all quality control samples met the reference requirements in the pooled QC sample. The trace element analysis workflow followed a previously published method [22].

The limit of detection (LOD) was calculated as 3 times the standard deviation (SD) of 10 consecutive measurements of the blank diluent. Values below the LOD were imputed as LOD/√2 and treated as continuous variables in the subsequent statistical analyses.

### 2.4. Thyroid Function Measurement

Thyroid function was assessed by measuring serum levels of thyroid-stimulating hormone (TSH), free thyroxine (FT4), and anti-thyroid peroxidase antibodies (TPOAb). These analyses were performed using the Architect system (Abbott Laboratories, Chicago, IL, USA) employing chemiluminescent microparticle immunoassay (CMIA) technology.

Quality control procedures were rigorously implemented to ensure data accuracy and precision. Each batch of samples included the analysis of internal quality control (IQC) materials provided by the manufacturer at two levels (normal and abnormal), as well as external quality assessment (EQA) samples from the National Center for Clinical Laboratories (Beijing, China). The inter-assay and intra-assay coefficients of variation (CVs) for all three analytes were maintained below 5% and 8%, respectively, meeting the manufacturer’s specifications and laboratory accreditation standards.

All reagents, calibrators, and controls were obtained from the corresponding Architect system (Abbott Laboratories, Chicago, IL, USA), used within their stated shelf life and according to the manufacturer’s protocols. This study’s diagnostic protocol follows WHO standards: TSH range 0.45–4.32 mIU/L and FT4 range 9.77–18.89 pmol/L [23]. Values outside these ranges were flagged and reviewed. Samples with hemolysis or significant lipemia were excluded from the analysis to avoid interference.

### 2.5. Covariates

Demographic information on the participants was collected at the first visit, including demographics, socioeconomic status, lifestyle factors, family history, and personal medical history. Age, body mass index (BMI), number of pregnancies, diabetes status, occupation type, education level and exercise frequency were included in the analysis as covariates. In the statistical analysis, age and BMI were treated as continuous variables, while all other covariates were analyzed as categorical variables. Smoking status and serum concentrations of Cd, Mn, Cu, and Zn were additionally included in the sensitivity analyses.

### 2.6. Statistical Analysis

Given the non-normal distribution of serum As and Hg concentrations, the data were natural log-transformed to reduce right skewness. No missing data were present for the variables included in the analysis. The association between As and Hg exposure and levels of TSH, FT4, and TPOAb was evaluated using multivariable linear regression analysis, with thyroid function indicators as the dependent variables and metal concentrations as the independent variables. To further characterize the shape of the dose–response relationship, dose–response relationships were assessed using restricted cubic splines (RCS) [24]. The number of knots (3–5) was selected based on the Akaike Information Criterion (AIC). Non-linearity was tested using the Wald test. The least absolute shrinkage and selection operator (LASSO) with L1 regularization was used for variable selection, and predictors were internally standardized. As and Hg exposures were penalized while covariates remained unpenalized. The optimal λ value was determined by 10-fold cross-validation, and variables with non-zero coefficients at λ.min were retained. Descriptive statistics are presented as mean ± SD for continuous variables and frequency (%) for categorical variables. Group comparisons were performed using Student’s *t*-test for continuous variables and Chi-square test for categorical variables. Sensitivity analyses were conducted by additionally adjusting for smoking status and co-exposure to Cd, Mn, Cu, and Zn. The primary covariate-adjusted model was retained as Model 1; Model 2 additionally included smoking status, and Model 3 further included Cd, Mn, Cu, and Zn. The non-linear association between Hg and FT4 was also re-evaluated using the extended covariate sets. Detailed methods are provided in Supplemental materials. All analyses were conducted in R version 4.5.0 using the packages *rcs* and *glmnet*.

### 2.7. TRAEC Strategy

To complement the epidemiological evidence and elucidate underlying mechanisms, the TRAEC strategy [25,26] was utilized to integrate in vivo and in vitro toxicological findings on As/Hg-induced thyroid toxicity. With the same literature search and duplicate removal as the meta-analysis, records were re-screened using new eligibility criteria for epidemiological, in vivo, and in vitro studies. The inclusion criteria were: (a) employed cohort, cross-sectional, case–control, in vivo, or in vitro study designs; (b) studies investigating the effect of As or Hg exposure on thyroid-related outcomes, including thyroid hormone levels, thyroid dysfunction, and thyroid disease; and (c) studies reporting sufficient methodological details to allow scoring according to the TRAEC framework. The exclusion criteria were: (a) duplicate publications, reviews, meta-analyses, conference abstracts, case reports, or guidelines; (b) studies with incomplete exposure or outcome information that precluded scoring; and (c) studies from which essential data could not be extracted for the 4 scoring dimensions (Reliability Scores, Weight of Concentrations, Risk Intensity, and Correlation Scores).

Two researchers independently extracted the following information from the full texts: title, authors, study period, study design, geographic location, population characteristics, detection methods, exposure concentrations, and exposure routes. In cases of disagreement, a third party was consulted for arbitration.

This study employed the TRAEC 1.1 strategy [17,18] to comprehensively evaluate the risk effects of As and Hg exposure on thyroid dysfunction. TRAEC strategy is a structured approach for assessing health risks associated with exposure to specific environmental chemicals. It involves the systematic collection of evidence from epidemiological, in vivo, and in vitro studies, followed by a quantitative evaluation of all studies based on four dimensions: Reliability Scores, Weight of Concentrations, Risk Intensity, and Correlation Scores. The results are ultimately integrated into a Comprehensive Evidence Score to classify the overall health risk level, thereby enabling a scientific and transparent assessment of the impacts of environmental chemicals. All evidence is independently scored by two researchers following TRAEC 1.1 Scoring Guidance. If a study combined two or more evidence types (e.g., epidemiological and in vivo), the scores for those types were aggregated accordingly. Scoring was performed using the TRAEC Web, a free online platform for TRAEC promotion and online calculation (https://traec.njmu.edu.cn/).

## 3. Results

### 3.1. Meta-Analysis of As and Hg on Thyroid Dysfunction Risk

Given the well-documented exposure prevalence and biological plausibility of As and Hg as thyroid disruptors alongside the conflicting findings in current epidemiological literature, this meta-analysis aimed to quantitatively synthesize the available evidence to derive a pooled estimate of the association between As/Hg exposure and the risk of thyroid dysfunction.

A total of 662 records were identified in PubMed and Web of Science based on the search strategy. After excluding 66 duplicate records, the remaining 596 records were further screened according to the inclusion and exclusion criteria. Full-text compliance assessments were conducted on the remaining 65 records, and 43 studies were ultimately excluded. Ultimately, 22 studies were included in the meta-analysis (Supplemental Figure S2).

**Figure 1:**
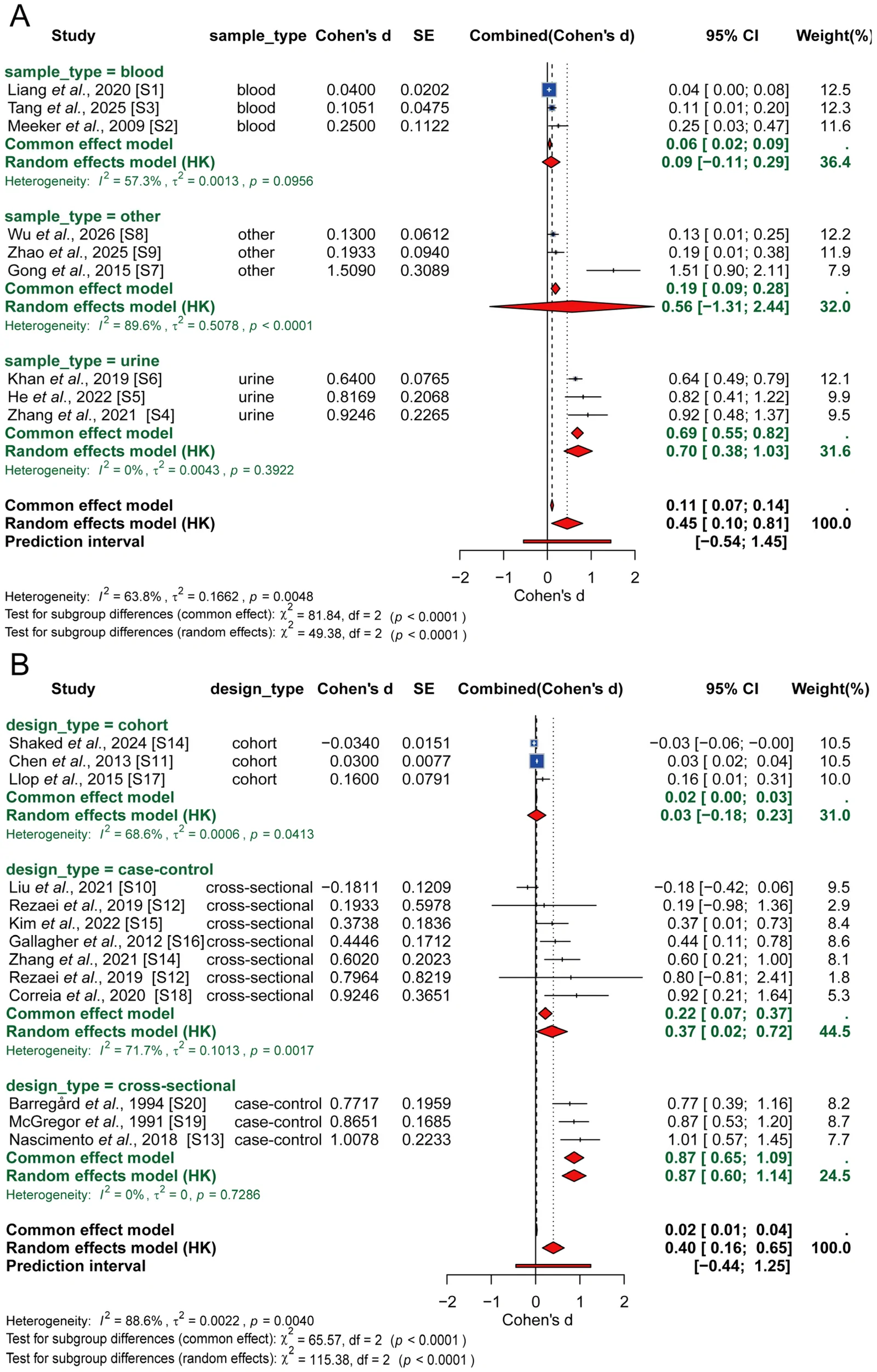
Forest plot of the association between As and Hg exposure and thyroid dysfunction. (**A**) Subgroup analysis by biospecimen type for As; (**B**) Subgroup analysis by study design for Hg. Blue squares represent study-specific effect estimates, with square size proportional to the study weight; the plus sign within each square indicates the point estimate, and horizontal lines indicate the corresponding 95% confidence intervals (CIs). Red diamonds represent pooled effect estimates, with their widths indicating the corresponding 95% CIs. The solid vertical line indicates the null effect (Cohen’s d = 0), whereas the dashed and dotted vertical lines indicate the overall estimates from the common-effect and random-effects models, respectively. Red horizontal lines at the bottom indicate the 95% prediction intervals. Green text indicates subgroup-level pooled estimates and heterogeneity statistics. Abbreviations: SE, standard error; 95% CI, 95% confidence intervals; HK, Hartung-Knapp.

Study quality was assessed using the Newcastle-Ottawa Scale (NOS). The mean NOS score was 7.15, indicating an overall high quality of the included literature (see Supplemental Table S2 for details). All studies were judged to provide adequate information for the analysis. The meta-analysis results for As and Hg exposure in relation to thyroid dysfunction risk are presented in Figure 1. For As exposure, a positive association with thyroid dysfunction risk was observed. The Cohen’s d from the random-effects model was 0.45 (95% CI: 0.10, 0.81), which was statistically significant. However, significant heterogeneity was detected among the studies (I^2^ = 63.8%, *p* = 0.0048), which was substantially moderated by the type of biological sample used for exposure assessment. Subgroup analysis revealed a strong and consistent positive association, specifically in studies using urine samples (pooled Cohen’s d = 0.70, 95% CI: 0.38, 1.03; I^2^ = 0%). Studies using blood or other samples showed positive but non-significant trends. A test for subgroup differences confirmed that the sample type was a key source of heterogeneity (*p* < 0.0001).

For Hg exposure, a positive association with thyroid dysfunction risk was also found, with a random-effects pooled effect size of 0.40 (95% CI: 0.16, 0.65). High heterogeneity was observed across the studies (I^2^ = 88.6%, *p* = 0.0040). Subgroup analysis by study design showed significant positive associations in both the cross-sectional group (pooled Cohen’s d = 0.37, 95% CI: 0.02, 0.72; I^2^ = 71.7%) and the case–control group (pooled Cohen’s d = 0.87, 95% CI: 0.60, 1.14; I^2^ = 0%), but not in the cohort group. Study design was identified as a significant moderator of heterogeneity (*p* < 0.0001). The Egger test results indicated that potential publication bias may exist only in the blood sample subgroup of the As subgroup (t = 48.09, *p* = 0.013). No significant bias was detected in other subgroups, and funnel plot analysis did not reveal significant publication bias (Supplemental Figure S3, Supplemental Table S3).

### 3.2. Association of As and Hg with Thyroid Hormone Levels in the Prospective Cohort

#### 3.2.1. Characteristics of the Study Population

Given that most evidence synthesized in the meta-analysis derived from cross-sectional investigations, causal inferences remain constrained. To overcome this shortcoming, we used data from a prospective cohort in Jiangsu including 800 adult women to evaluate the impact of As and Hg exposure on thyroid dysfunction, thereby establishing temporality and strengthening etiological evidence. Detailed information was collected on demographic characteristics, thyroid function, related conditions, and lifestyle factors. The overall characteristics of the study participants are presented in Table 1.

#### 3.2.2. Concentrations of As and Hg in the Cohort

We measured serum As and Hg concentrations in participants during their first visit. The results show that As and Hg were both detected in nearly all serum samples from the cohort. The distributions are summarized in Table 2.

#### 3.2.3. Association Between As and Hg Concentrations and Thyroid Hormones

**Figure 2:**
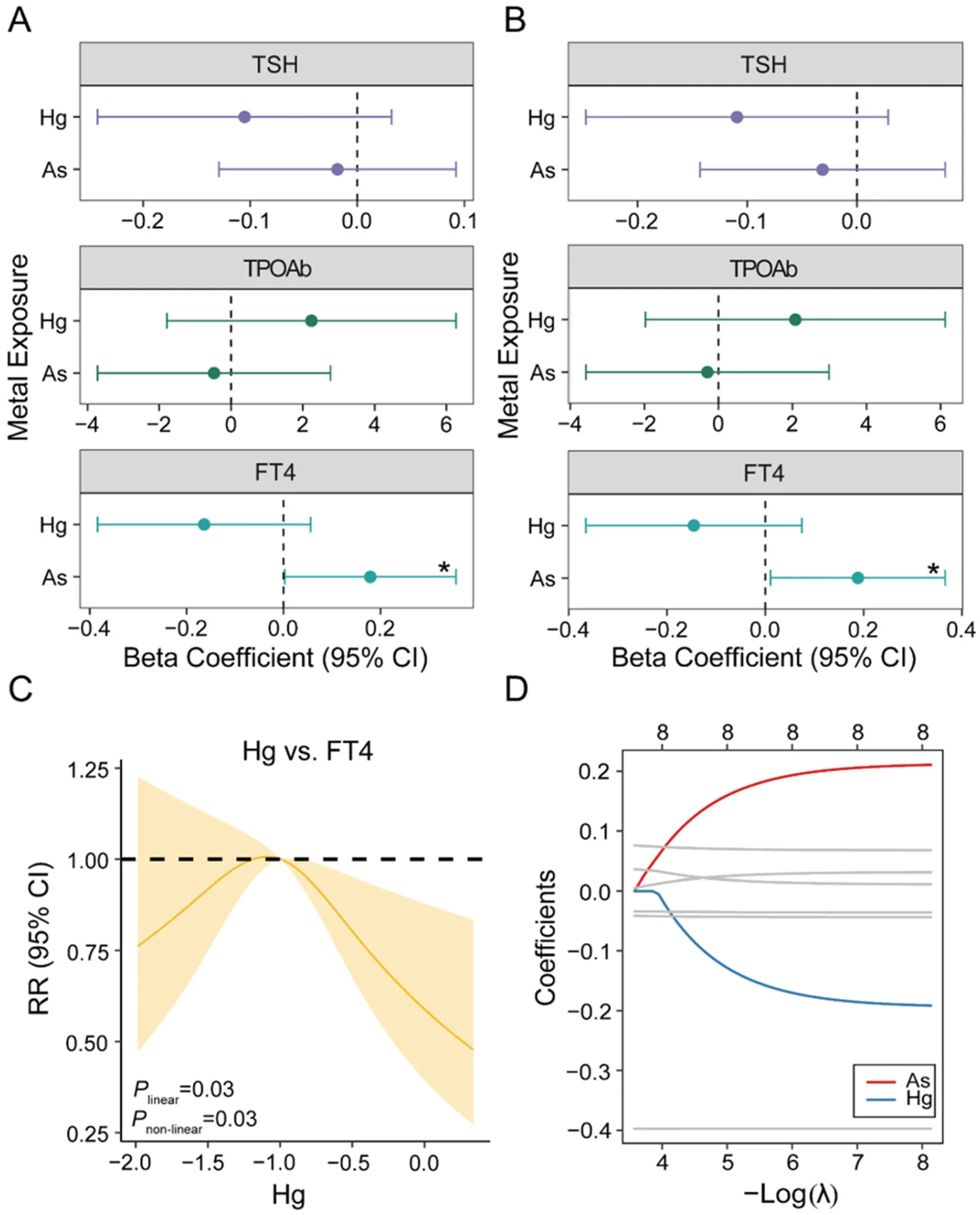
Potential associations of As and Hg exposure and thyroid hormone levels. (**A**) Unadjusted associations between As and Hg with TSH, TPOAb and FT4 in the cohort; (**B**) Adjusted associations between As and Hg with TSH, TPOAb and FT4 in the cohort; Age, BMI, number of pregnancies, diabetes status, occupational type, education level, exercise frequency were adjusted in the analysis. In panels A and B, the points represent β coefficients, the horizontal lines represent their 95% CIs, and the vertical dashed lines indicate the null value (β = 0); (**C**) Dose–response relationship between Hg and FT4 in the cohort. The orange curve represents the estimated dose–response relationship, the yellow shaded area represents the corresponding 95% CI, and the horizontal dashed line indicates the null value (RR = 1); (**D**) Coefficient shrinkage paths for FT4-related variables in the cohort; Each curve represents the coefficient of a predictor variable as a function of the -log(λ) (the negative logarithm of λ). The red and blue curves represent the coefficient paths for As and Hg, respectively, whereas the grey curves represent those for the other predictor variables included in the model. Significant associations are indicated by * (*p*  <  0.05). Abbreviations: As, arsenic; Hg, mercury; TSH, thyroid-stimulating hormone; FT4, free thyroxine; TPOAb, anti-thyroid peroxidase antibody; 95% CI, 95% confidence intervals; RR, relative risk.

Figure 2A,B present the associations between log-transformed As and Hg exposure and thyroid function indicators, derived from generalized linear models (GLM) in the cohort. The detailed results are provided in Supplemental Table S4.

In the adjusted model, As was positively associated with FT4 levels (β = 0.19, 95% CI: 0.01, 0.37). This association was consistent in both unadjusted and adjusted analyses, suggesting a robust relationship independent of confounding factors. However, Hg showed a negative, albeit non-significant, trend with FT4 levels (β = −0.15, 95% CI: −0.37, 0.07). Neither As nor Hg exposure was significantly associated with TSH or TPOAb levels.

Given that linear models may mask complex relationships, we further conducted RCS regression to characterize the dose–response shape between Hg and FT4. Figure 2C presents that a significant non-linear association was observed between Hg exposure and FT4 levels (*p* for non-linearity = 0.03). Hg showed an inverted U-shaped association with FT4, indicating a biphasic dose-dependent effect on thyroid function: potential stimulation at low exposure levels and inhibition at higher levels after a threshold concentration. Further dose–response results are presented in Supplemental Figure S4.

Building on the previous GLM results, LASSO regression was employed for variable selection, focusing on FT4 in the cohort. The corresponding coefficient paths are shown in Figure 2D. Under the minimum error criterion (λ.min), As (β = 0.21) and Hg (β = −0.19) were included in the final FT4 model, confirming their independent predictive roles for FT4 variation in this cohort. Cross-validation curves for the LASSO regression are provided in Supplemental Figure S5.

Sensitivity analyses showed that additional adjustment for smoking status and co-exposure to Cd, Mn, Cu, and Zn did not materially alter the findings. The positive association between As and FT4 and the nonlinear association between Hg and FT4 remained statistically significant (Supplemental Tables S5–S7 and Supplemental Figure S6).

### 3.3. Thyroid Risk Assessment Using the TRAEC Strategy

Although the epidemiological findings from the meta-analysis and cohort study provide consistent evidence linking As and Hg exposure to thyroid dysfunction in human populations, the underlying biological mechanisms and causal pathways remain incompletely characterized. To bridge this gap between population-level associations and molecular-level causation, the TRAEC strategy was employed to supplement the existing epidemiological evidence with additional in vivo and in vitro studies. Starting from the 596 records retained after duplicate removal in the meta-analysis, we applied a new set of inclusion and exclusion criteria to select all eligible evidence. Full-text eligibility assessment was conducted on the remaining 73 records, resulting in the exclusion of 28 studies. Ultimately, 45 studies from literature together with the present cohort study were included in the TRAEC-based risk assessment (Supplemental Figure S7).

The final set of 46 studies comprised 23 epidemiological studies (Supplemental Table S8), 18 in vivo studies (Supplemental Table S9), and 5 in vitro studies (Supplemental Table S10). Most studies evaluated the effects of As or Hg exposure on thyroid health outcomes, mainly thyroid dysfunction and thyroid hormone levels. Among them, 26 focused on As exposure, 22 on Hg exposure, and 2 on combined As-Hg exposure. All included studies focused on thyroid-related outcomes, including specific thyroid dysfunction and thyroid hormone levels.

#### 3.3.1. Epidemiological Studies

A summary of the included epidemiological studies is presented in Supplemental Table S8. The 23 studies consisted of 12 cross-sectional studies, 5 case–control studies, and 6 cohort studies. Sample sizes ranged from 32 to 7688 participants, with a median of 394, suggesting that most included studies had sufficient power to detect meaningful associations. Exposure assessment mainly focused on environmental matrices (drinking water and food consumption) and biological samples (urine, blood, and hair). Blood As concentrations (44.4% of studies, median range: 2.27–17.12 ng/mL) and blood Hg concentrations (66.7%, 0.53–22.1 ng/mL) were the key exposure biomarkers. A total of 17 studies (73.9%) reported the effects of As or Hg exposure on thyroid dysfunction, including clinical and subclinical hyperthyroidism, hypothyroidism, and autoimmune thyroiditis. The remaining 6 studies (26.1%) explored the associations between As or Hg exposure and risks of goiter, thyroid nodules, and thyroid cancer.

Among the 23 included studies, 7 investigated the association between As exposure and thyroid hormone levels (TSH and FT4), with 6 reporting significant relationships. For example, Liang et al. found no significant association between maternal As exposure and maternal FT4 levels (β = −0.05, 95% CI: −0.22, 0.11) but identified a significant negative correlation with neonatal FT4 (β = −0.22, 95% CI: −0.42, −0.02) [27]. Two additional studies by Zhang et al. and He et al. reported significant associations between As exposure and thyroid cancer [15,28]. Regarding Hg exposure, the evidence was less consistent. Ten studies suggested an increased risk of thyroid dysfunction with Hg exposure. For instance, Kim et al. reported a positive association between elevated urinary Hg and thyroid cancer risk (OR = 1.97, 95% CI: 1.03, 3.80) [29]. In contrast, Shaked et al. observed a negative correlation between Hg exposure and thyroid cancer risk [14]. Additionally, three other studies found no significant association between Hg exposure and thyroid outcomes.

#### 3.3.2. In Vivo Studies

Among the 18 in vivo studies (Supplemental Table S9), 13 utilized mouse, rat, or zebrafish models to assess As- or Hg-induced thyroid toxicity, while the remaining 5 employed other models such as goats, rabbits, and guinea pigs. All studies adopted single-exposure designs, administering varying concentrations of As or Hg compounds (primarily inorganic arsenic and mercury chloride) via drinking water, feed, or oral gavage over periods ranging from 3 weeks to 6 months. In vivo endpoints mainly covered serum thyroid hormones and thyroid histopathology.

Most existing in vivo studies (17 out of 18) have reported significant thyroid-disrupting effects following As or Hg exposure. For instance, Fan et al. and Guzzolino et al. found that chronic As and Hg exposure disrupted thyroid hormone synthesis and altered protein expression in thyroid tissue [30,31]. Nevertheless, Mukhi et al. reported inconsistent findings in their research. In their experiment, Wistar rats were daily given As_2_O_3_ or HgCl_2_ by oral gavage and thyroid hormone levels were determined after continuous exposure. Their results showed that As exposure did not significantly alter thyroid function indicators compared with the control group. By comparison, Hg exposure markedly affected thyroid function, significantly reducing serum Triiodothyronine (T3) and Thyroxine (T4) levels while elevating TSH levels [32].

Based on available in vivo evidence, the mechanisms by which As and Hg induce thyroid damage primarily converge on two common pathways: transcriptional dysregulation of HPT axis-related genes and the resulting hormonal imbalance (decreased T3/T4, compensatory increase in TSH), as well as the activation of the oxidative stress-inflammation cascade (ROS/NF-κB/NLRP3), which subsequently leads to apoptosis, pyroptosis, and fibrotic remodeling of thyroid follicular cells.

#### 3.3.3. In Vitro Studies

Details regarding the specific compounds, doses, and exposure durations across studies are summarized in Supplemental Table S10. Briefly, these investigations employed human thyroid cell lines, including normal thyroid epithelial cells (Nthy-ori-3-1), medullary thyroid carcinoma cells (TT), and differentiated thyroid carcinoma cells (FTC-238 and CGTH-W-1). Assessed endpoints included cell viability, apoptosis, proliferation, differentiation status, iodine uptake capacity, and key signaling pathways. Exposure concentrations ranged from 0.1 to 100 µM for inorganic arsenic and from 0.1 to 20 µM for methylmercury, with durations varying between 24 h and 18 days.

Fan et al. observed NaAsO_2_-induced mitochondrial apoptosis in thyroid cells, whereas Maggisano et al. found that low doses of methylmercury were associated with thyroid cell proliferation [33,34]. Additionally, Fröhlich et al. demonstrated that arsenic trioxide reduced proliferation, increased iodide uptake, and induced apoptosis in transformed human thyroid cells, highlighting the pleiotropic effects of As on thyroid cell biology [35]. A broader in vitro screening study by Costa et al. across multiple cell types also identified the thyroid as a potential target of methylmercury toxicity, though without elucidating underlying mechanisms [36]. Taken together, these five in vitro studies point to two distinct mechanisms of thyroid cell toxicity: inorganic arsenic predominantly triggers mitochondrial apoptosis through dysregulation of the Bax/Bcl-2 ratio, whereas low-dose methylmercury activates the ERK signaling cascade to stimulate cellular proliferation.

#### 3.3.4. Evidence Scoring

**Figure 3:**
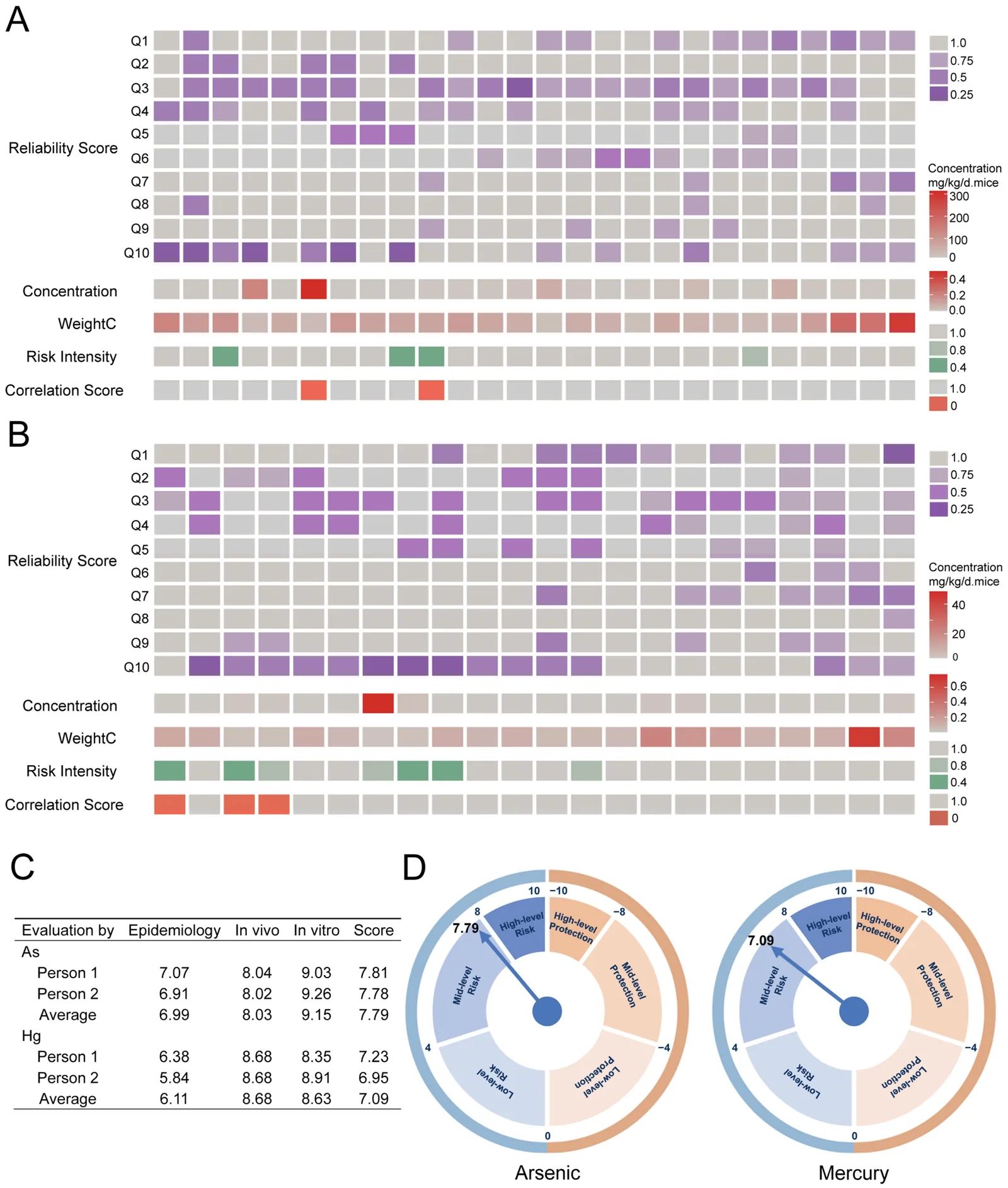
Risk assessment with all the evidence based on the TRAEC strategy. (**A**) Reliability scores, correlation scores, and risk intensity for As; (**B**) Reliability scores, correlation scores, and risk intensity for Hg; (**C**) Epidemiological studies, in vivo studies, in vitro studies, and comprehensive evidence scores; (**D**) Composite evidence scores for all the studies. Abbreviations: As, arsenic; Hg, mercury.

Integrating 23 epidemiological studies, 18 in vivo studies, and 5 in vitro studies, evidence scoring was completed based on four dimensions: Reliability Scores, Weight of Concentrations, Risk Intensity, and Correlation Scores. The scoring results show that reviewers assigned relatively moderate scores to epidemiological studies on As (average 6.99), while in vivo studies (8.03) and in vitro studies (9.15) received higher scores, with in vitro studies scoring the highest. For Hg, epidemiological studies received lower scores (average 6.11), in vivo studies received higher scores (8.68), and in vitro studies also scored at a high level (8.63). Results at the category level indicate that As demonstrated the strongest effect in in vitro studies (9.15), while Hg showed the most significant effect in in vivo studies (8.68). Scores for both metal(loid)s in epidemiological studies were lower than those in in vivo and in vitro studies. The Comprehensive Evidence Score showed that both As (7.79) and Hg (7.09) were at a moderate level, suggesting potential thyroid-related health concerns associated with As and Hg exposure (Figure 3).

## 4. Discussion

Despite growing evidence linking As and Hg exposure to thyroid dysfunction, key questions regarding causality and integrated risk quantification remain unresolved. In this study, we first conducted a meta-analysis and found that both As and Hg exposure were significantly associated with an increased risk of thyroid dysfunction. Subsequently, using prospective cohort data from Jiangsu, we systematically investigated the effects of As and Hg exposure on thyroid function. Multiple statistical methods, including generalized linear models, dose–response analysis, and LASSO regression, indicated that As exposure was linearly and positively associated with elevated FT4 levels, whereas Hg exposure showed a significant non-linear (inverted U-shaped) association with FT4. Based on these findings, the TRAEC framework was applied to provide a comprehensive risk score, resulting in scores of 7.79 for As and 7.09 for Hg, both corresponding to a moderate risk level. These results indicate that As and Hg exposure may be associated with altered FT4 levels, although no significant associations were observed for TSH or TPOAb. To quantitatively analyze the effects of As and Hg on thyroid dysfunction, we conducted a meta-analysis by synthesizing existing epidemiological studies. The results indicate that existing evidence supports a positive correlation between As/Hg exposure and thyroid dysfunction. Consistent with these findings, a meta-analysis by Hu et al. found a significant association between Hg exposure and thyroid hormone levels [37]; however, a meta-analysis by van Gerwen et al. on heavy metals and thyroid cancer did not find that exposure to As or Hg contributes to the development of thyroid cancer [5]. This discrepancy is likely attributable to differences in the specific thyroid endpoints examined. Notably, meta-analysis is limited to epidemiological evidence and cannot elucidate underlying mechanistic pathways or fully characterize risk beyond population-level health associations.

Most of the existing evidence comes from cross-sectional studies, making it difficult to infer a causal relationship. Therefore, we continued a prospective cohort study among 800 adult women in Jiangsu, a region with low but detectable As and Hg exposure levels. The cohort findings indicated that As and Hg exposure was associated with alterations in thyroid hormone levels, particularly FT4. This finding aligns with previous studies on low-dose exposure [6]. Notably, although Hg exposure did not exhibit a significant linear effect on FT4 in the GLM framework, dose–response analysis using RCS revealed a significant non-linear relationship, characterized by an inverted U-shaped curve. This pattern suggests that low Hg exposure may mildly stimulate thyroid hormone synthesis or release, while higher exposure levels may exert inhibitory effects, possibly through oxidative stress, disruption of iodine metabolism, or interference with thyroid hormone synthesis and transport pathways. Collectively, our prospective cohort findings provide robust temporal evidence supporting the thyroid-disrupting potential of low-level As and Hg exposure in adult women, with distinct association patterns for the two metal(loid)s. These results underscore the need for continued surveillance of environmental metal(loid) exposure and its subtle endocrine consequences, even in regions with relatively low contamination levels.

Numerous in vivo and in vitro studies have demonstrated the effects of As and Hg on thyroid health. An animal study on SD rats reported that arsenite exposure activates the TLR4/NF-κB-mediated inflammatory response in thyroid tissue, leading to thyroid dysfunction [38]. In zebrafish embryos, Hg exposure disrupts the expression of genes involved in thyroid hormone synthesis, transport, and metabolism, resulting in abnormal T4/T3 levels and developmental malformations, with effects more potent than cadmium [31]. In vitro studies further demonstrate that low doses of methylmercury induced the proliferation of thyroid cells through modulation of the ERK pathway, suggesting a potential tumorigenic risk [34].

Although numerous studies have confirmed the toxicity of As and Hg to the thyroid, significant heterogeneity in study populations, exposure levels, study types, and study designs makes it difficult to conduct a unified quantitative risk assessment across epidemiological, in vivo, and in vitro studies. Therefore, we adopted the TRAEC strategy. In a prior study, Ning et al. assessed consistency using four tools (ToxRTool, SciRAP, OHAT, RoB, and IRIS) [25]. Their findings confirmed the robustness of the conclusions derived from the TRAEC framework and demonstrated high consistency with existing assessment tools. Furthermore, the increasing number of risk assessment studies adopting the TRAEC strategy validates the reliability and effectiveness of this framework in assessing the toxicological risks of target chemicals [18,39].

Collectively, the TRAEC strategy offers a standardized framework for integrating epidemiological and experimental evidence. Applying it to As and Hg yielded risk scores of 7.79 and 7.09, respectively, placing both in the moderate-risk range under the TRAEC framework. Given the weighting of environmentally relevant evidence and the observation of thyroid dysfunction across different exposure levels, these findings constitute a robust warning. Despite strict regulations on As and Hg, their persistent detectability calls for their inclusion in public health monitoring and further refinement of risk assessment methodologies [40,41].

This study has several limitations. First, substantial heterogeneity was observed in the meta-analysis, particularly for Hg. Although subgroup analyses identified biospecimen type and study design as potential sources of heterogeneity, differences in study populations, exposure assessment methods, exposure levels, and thyroid-related outcomes may also have contributed to the observed variability. Therefore, the pooled estimates should be interpreted with caution. Second, thyroid assessment was restricted to TSH, FT4, and TPOAb; broader profiling (e.g., TT3, FT3, TgAb) was not performed, potentially limiting detection of subtle or atypical dysfunction. Although additional adjustment for smoking status and co-exposure to Cd, Mn, Cu, and Zn did not materially alter the findings, information on iodine and selenium status, alcohol consumption, dietary patterns, particularly seafood intake, and exposure to other endocrine-disrupting chemicals was unavailable. Therefore, residual confounding from these unmeasured factors cannot be excluded. Third, due to the lack of toxicokinetic data, no standardized dose-conversion factors were available for in vitro studies, hindering direct extrapolation to human exposure scenarios. Similarly, differences between animal models and humans in thyroid physiology, toxicokinetics, exposure routes, and administered doses limit the direct extrapolation of in vivo findings to human populations. Therefore, the experimental evidence should primarily be interpreted as supporting biological plausibility rather than establishing definitive human health effects. Finally, in vitro research on the thyroid toxicity of As and Hg has remained sparse in recent years, limiting the depth of mechanistic insight available for risk interpretation.

## 5. Conclusions

Meta-analysis revealed the adverse effects of As and Hg on the onset and progression of thyroid dysfunction. Findings from the prospective cohort further indicated that As exposure was linearly and positively associated with FT4 levels, while dose–response analysis revealed a significant non-linear, inverted U-shaped association between Hg and FT4; however, no significant associations were observed for TSH or TPOAb. Based on integrated epidemiological, in vivo, and in vitro evidence, we systematically evaluated the thyroid dysfunction risk associated with As and Hg exposure via the TRAEC strategy, with overall risk scores of 7.79 and 7.09, respectively. Taken together, findings from the meta-analysis, prospective cohort study, and integrated epidemiological, in vivo, and in vitro evidence consistently indicate that As and Hg exposure is associated with thyroid dysfunction, while highlighting distinct exposure–response patterns for the two metal(loid)s and supporting their prioritization in environmental health monitoring and thyroid-related risk assessment.

## Figures and Tables

**Table 1:** Demographic Characteristics in the cohort (*N* = 800).

Characteristics	*N* (%) or Mean ± SD
TSH (μIU/mL)	2.14 ± 0.95
FT4 (pmol/L)	12.69 ± 1.52
TPOAb (IU/mL)	22.00 ± 27.83
Age (years)	29.53 ± 3.60
BMI (kg/m^2^)	21.93 ± 3.33
Number of pregnancies	
0	412 (51.5)
≥1	388 (48.5)
Diabetes status	
Yes	135 (16.9)
No	665 (83.1)
Occupational type	
Administrative work	585 (73.1)
General work	156 (19.5)
Housework	59 (7.4)
Education level	
Middle school	45 (5.6)
University graduate	624 (78)
Postgraduate or above	131 (16.4)
Exercise frequency	
None	231 (28.9)
Once per week	256 (32.0)
Two to three times per week	129 (16.1)
Four times per week	64 (8.0)
≥5 times per week	120 (15.0)

Abbreviations: TSH, thyroid-stimulating hormone; FT4, free thyroxine; TPOAb, anti-thyroid peroxidase antibody; BMI, body mass index; SD, standard deviation.

**Table 2:** Serum As and Hg concentrations in the cohort (µg/L).

Elements	LOD	>LOD (%)	Mean	SD	Percentile			Range
					25	50	75	(2.5–97.5%)
As	0.14	99	0.80	0.72	0.40	0.61	0.93	0.20–2.75
Hg	0.05	100	0.40	0.25	0.25	0.34	0.47	0.13–1.02

Abbreviations: LOD, limit of detection; SD, standard deviation; As, arsenic; Hg, mercury.

## Data Availability

The original contributions presented in this study are included in the article/Supplementary Material. Further inquiries can be directed to the corresponding authors.

## Supplementary Materials

The following supporting information can be downloaded at https://www.mdpi.com/article/10.3390/toxics14090795/s1: Figure S1: Flowchart of the participants selection in the study; Figure S2: Flow diagram of selecting articles for meta-analysis; Figure S3: Funnel plots for publication bias assessment in meta-analyses on heavy metals and thyroid disorders; Figure S4: Dose–response relationship between As and Hg exposure and thyroid hormone levels; Figure S5: Cross-validation curve of FT4-related variables in the cohort; Figure S6: Hg–FT4 restricted cubic spline sensitivity analysis after additional covariate adjustment; Figure S7: Flow diagram of selecting articles for TRAEC-based risk assessment; Table S1: Detailed search strategy; Table S2: Detailed Evaluation of Literature Quality Based on the NOS Scale for Studies Included in the Meta-Analysis; Table S3: Egger test results for the As and Hg subgroup analysis; Table S4: Association between heavy metals and maternal thyroid hormone levels (μg/L); Table S5: Associations of As and Hg with FT4 after additional adjustment for smoking status and co-exposure to other metals; Table S6: Hg–FT4 restricted cubic spline sensitivity analysis after additional covariate adjustment; Table S7: Spearman correlations among As, Hg, and other metals; Table S8: The contextual details of included epidemiological studies; Table S9: The contextual details of included in vivo studies; Table S10: The contextual details of included in vitro studies; Supplementary Methods: Additional details on the study population, participant selection, ethical approval, sample collection, and sensitivity analyses. References [14,15,27,28,29,30,32,33,34,35,36,38,42,43,44,45,46,47,48,49,50,51,52,53,54,55,56,57,58,59,60,61,62,63,64,65,66,67,68,69,70,71] cited in Supplementary Materials.

## Abbreviations

The following abbreviations are used in this manuscript:

AIC	Akaike information criterion
As	Arsenic
BMI	Body mass index
CI	Confidence interval
CMIA	Chemiluminescent microparticle immunoassay
CV	Coefficient of variation
EQA	External quality assessment
ERK	Extracellular signal-regulated kinase
FT3	Free triiodothyronine
FT4	Free thyroxine
GLM	Generalized linear model
Hg	Mercury
HK	Hartung–Knapp method
HPT	Hypothalamic–pituitary–thyroid
HR	Hazard ratio
ICP-MS	Inductively coupled plasma mass spectrometry
IQC	Internal quality control
IRIS	Integrated Risk Information System
LASSO	Least absolute shrinkage and selection operator
LOD	Limit of detection
NF-κB	Nuclear factor kappa B
NLRP3	NLR family pyrin domain-containing protein 3
NOS	Newcastle–Ottawa Scale
OHAT	Office of Health Assessment and Translation
OR	Odds ratio
PRISMA	Preferred Reporting Items for Systematic Reviews and Meta-Analyses
QC	Quality control
RCS	Restricted cubic splines
RoB	Risk of bias
ROS	Reactive oxygen species
RR	Relative risk
RSD	Relative standard deviation
SciRAP	Science in Risk Assessment and Policy
SD	Standard deviation
SE	Standard error
T3	Triiodothyronine
T4	Thyroxine
TgAb	Thyroglobulin antibody
TLR4	Toll-like receptor 4
ToxRTool	Toxicological Data Reliability Assessment Tool
TPOAb	Anti-thyroid peroxidase antibody
TRAEC	Targeted Risk Assessment for Environmental Chemicals
TSH	Thyroid-stimulating hormone
TT3	Total triiodothyronine
WHO	World Health Organization
