# Supplementary material for: Effects of Arsenic and Mercury Exposure on Thyroid Dysfunction: An Evidence-Based Risk Assessment Using the TRAEC Strategy
Source: Toxics. 2026 Sep 8;14(9):795. doi: 10.3390/toxics14090795 (PMC13611428; doi:10.3390/toxics14090795)
Supplement: Supplementary file 1 [file toxics-14-00795-s001.zip › toxics-4528844-supplementary.pdf]

Effects of arsenic and mercury exposure on thyroid dysfunction: an evidence-based risk assessment using the TRAEC strategy

Supplementary materials

The Contents

Supplemental Table S1. Detailed search strategy 3

Supplemental Table S2. Detailed Evaluation of Literature Quality Based on the NOS Scale for Studies Included in the Meta-Analysis 4

Supplemental Table S3. Egger test results for the As and Hg subgroup analysis 6

Supplemental Table S4. Association between heavy metals and maternal thyroid hormone levels (μg/L) 7

Supplemental Table S5. Associations of As and Hg with FT4 after additional adjustment for smoking status and co-exposure to other metals 8

Supplemental Table S6. Hg-FT4 restricted cubic spline sensitivity analysis after additional covariate adjustment 9

Supplemental Table S7. Spearman correlations among As, Hg, and other metals 10

Supplemental Table S8. The contextual details of included epidemiological studies 11

Supplemental Table S9. The contextual details of included *in vivo* studies 13

Supplemental Table S10. The contextual details of included *in vitro* studies 14

Supplemental Figure S1. Flowchart of the participants selection in the study 15
Supplemental Figure S2. **Flow diagram of selecting articles for meta-analysis** 16
Supplemental Figure S3. Funnel plots for publication bias assessment in meta-analyses on heavy metals and thyroid disorders. 17
Supplemental Figure S4. Dose–response relationship between As and Hg exposure and thyroid hormone levels 18
Supplemental Figure S5. Cross-validation curve of FT4-related variables in the cohort 19
Supplemental Figure S6. Hg–FT4 restricted cubic spline sensitivity analysis after additional covariate adjustment 20
Supplemental Figure S7. **Flow diagram of selecting articles for TRAEC-based risk assessment** 21
Methods and materials supplementation 22

Supplemental Table S1. Detailed search strategy

Database	Search strategy	Numbers
**Pubmed**	((Mercury) OR (Mercury Compounds) OR (Mercury Poisoning)) AND ((((Thyroid Diseases) OR (Thyroid Function Tests) OR (Thyreotropin) OR (TSH) OR (Thyroid Stimulating Hormone))))	158
	((Arsenic Exposure) OR (Arsenic Poisonings) OR (Arsenical Neurotoxicity Syndrome) OR (Arsenic Encephalopathy) OR (Inorganic Arsenic Poisonings) OR (Arsenic Poisoning, Organic)) AND ((Thyroid Diseases) OR (Thyroid Function Tests) OR (Thyreotropin) OR (TSH) OR (Thyroid Stimulating Hormone))	60
**Web of Science**	(TS=("Mercury Poisoning") OR TS=("Methylmercury") OR TS=("Ethylmercury") OR TS=("Mercuric Chloride") OR TS=(Mercury)) AND (TS=("Thyroid Diseases") OR TS=("Thyroid Function Tests") OR TS=(Thyreotropin) OR TS=(TSH) OR TS=("Thyroid Stimulating Hormone"))	319
	(TS=("Arsenic Exposure") OR TS=("Arsenic Poisonings") OR TS=("Arsenical Neurotoxicity Syndrome") OR TS=("Arsenic Encephalopathy") OR TS=("Inorganic Arsenic Poisonings") OR TS=("Arsenic Poisoning, Organic")) AND (TS=("Thyroid Diseases") OR TS=("Thyroid Function Tests") OR TS=(Thyreotropin) OR TS=(TSH) OR TS=("Thyroid Stimulating Hormone"))	125

Supplemental Table S2. Detailed Evaluation of Literature Quality Based on the NOS Scale for Studies Included in the Meta-Analysis

**Cohort**	**Selection**				**Comparability**	**Outcome**			Scores
	Representativeness	Selection	Ascertainment	Demonstration	Comparability	Assessment	Was	Adequacy	
Liang et al., 2020	★	★	★	★	★★	★	★	★	9
Wu et al., 2025	★	★	★	★	★★	★	★	-	8
Nascimento et al., 2018	★	★	★	-	★★	★	-	-	6
Kim et al., 2022	★	★	★	★	★★	★	★	★	9
Llop et al., 2015	★	★	★	★	★★	★	★	-	8
**Case–Control**	**Selection**				**Comparability**	**Outcome**			Scores
	Adequacy	Representativeness	Selection	Definition	Comparability	Ascertainment	Same	Non-reported	
Tang et al., 2025	★	-	★	★	★★	★	★	-	7
Zhang et al., 2020	★	-	★	★	★★	★	★	-	7
He et al., 2022	★	-	★	★	★★	★	★	-	7
Zhao et al., 2025	★	★	★	★	★★	★	★	★	9
Liu et al.., 2021	★	★	★	★	★★	★	★	-	8
**Cross-Sectional**	**Selection**				**Comparability**	**Outcome**			Scores
	Representativeness	Sampling	Sample	Non-respondents	Comparability	Assessment	Statistical	Response	
Meeker et al., 2009	-	-	★	-	★★	★	★	-	5
Khan et al.., 2019	★	★	-	-	★★	★	★		6
Gong et al., 2015	★	★	★	-	★★	★	★	-	7
Chen et al., 2013	★	★	★	-	★★	★	★	★	8

Rezaei et al., 2019	★	-	★	-	★★	★	★	-	6
Shaked et al., 2024	★	★	★	-	★★	★	★	-	7
Gallagher et al., 2012	★	★	★	-	★★	★	★	★	8
Correia et al., 2020	★	-	★	-	★★	★	★	-	6
McGregor & Mason, 1991	★	-	★	-	★★	★	★	-	6
Barregård et al., 1994	★	-	★	-	★★	★	★	-	6

Supplemental Table S3. Egger test results for the As and Hg subgroup analysis

**As(Sample_type)**	**t**	**df**	**Bias estimate(SE)**	***P***
Blood	48.09	1	2.3086(0.0480)	0.0132
Urine	5.46	1	1.6461(0.3016)	0.1154
Other	4.13	1	5.2289(1.2676)	0.1514
**Hg(Design_type)**				
Cross-sectional	1.51	5	2.0349(1.3449)	0.1907
Cohort	-0.02	1	-0.0748(4.2664)	0.9888
Case–control	0.59	1	2.0485(3.4430)	0.6583

Abbreviations: SE, standard error; As, arsenic; Hg, mercury.

Supplemental Table S4. Association between heavy metals and maternal thyroid hormone levels (μg/L)

Elements	TSH(μIU/mL)	FT4(pmol/L)	TPOAb(IU/mL)
Unadjusted			
As	-0.02 (-0.13, 0.09)	0.18(0.002, 0.36) *	-0.48(-3.72, 2.77)
Hg	-0.11(-0.24, 0.03)	-0.16(-0.38, 0.06)	2.24(-1.79, 6.26)
Adjusted			
As	-0.03(-0.14, 0.08)	0.19(0.01, 0.37) *	-0.30(-3.58, 2.98)
Hg	-0.11(-0.25, 0.03)	-0.15(-0.37, 0.07)	2.08(-1.97, 6.13)

Abbreviations: TSH, thyroid-stimulating hormone; FT4, free thyroxine; TPOAb, anti-thyroid peroxidase antibody.

The models were adjusted for age, BMI level, number of pregnancies, diabetes status, occupational type, education level, exercise frequency.

*$P < 0.05$

Supplemental Table S5. Associations of As and Hg with FT4 after additional adjustment for smoking status and co-exposure to other metals.

Exposure	Model	β	95% CI	*P* value	N	Change in β*
As	Model 1	0.192	0.01 to 0.37	0.034	800	Reference
As	Model 2	0.194	0.02 to 0.37	0.032	800	1.1%
As	Model 3	0.225	0.04 to 0.41	0.018	800	17.4%
Hg	Model 1	-0.150	-0.37 to 0.07	0.181	800	Reference
Hg	Model 2	-0.147	-0.37 to 0.07	0.189	800	1.9%
Hg	Model 3	-0.160	-0.39 to 0.06	0.162	800	7.3%

Abbreviations: CI, confidence interval; FT4, free thyroxine; As, arsenic; Hg, mercury.

Model 1 was adjusted for age, BMI, number of pregnancies, diabetes status, occupational type, education level, and exercise frequency. Model 2 was additionally adjusted for smoking status. Model 3 was additionally adjusted for Cd, Mn, Cu, and Zn. Change in β represents the absolute percentage change relative to Model 1: $|(\beta\text{Model} - \beta\text{Model 1})/\beta\text{Model 1}| \times 100\%$.

Supplemental Table S6. Hg-FT4 restricted cubic spline sensitivity analysis after additional covariate adjustment.

Model	Covariate specification	N	*P* overall	*P* for non-linearity	Reference Hg	Knot locations
Model 1	Primary covariate-adjusted model	800	0.034	0.029	-0.996	-1.619, -0.996, -0.440
Model 2	Model 1 + smoking	800	0.032	0.026	-0.996	-1.619, -0.996, -0.440
Model 3	Model 2 + Cd + Mn + Cu + Zn	800	0.036	0.032	-0.996	-1.619, -0.996, -0.440

Abbreviations: FT4, free thyroxine; Hg, mercury; N, number of participants; Cd, cadmium; Mn, manganese; Cu, copper; Zn, zinc.

Model 1 was adjusted for age, BMI, number of pregnancies, diabetes status, occupational type, education level, and exercise frequency. Model 2 was additionally adjusted for smoking status. Model 3 was additionally adjusted for Cd, Mn, Cu, and Zn. All models used three knots and the same median Hg concentration as the reference. Hg concentrations were analyzed on the natural log-transformed scale.

Supplemental Table S7. Spearman correlations among As, Hg, and other metals.

	As	Hg	Cd	Mn	Cu	Zn
**As**	1.000	0.151	0.194	0.223	-0.036	0.181
**Hg**	0.151	1.000	0.139	0.180	0.106	0.146
**Cd**	0.194	0.139	1.000	0.381	0.097	0.228
**Mn**	0.223	0.180	0.381	1.000	0.074	0.390
**Cu**	-0.036	0.106	0.097	0.074	1.000	0.257
**Zn**	0.181	0.146	0.228	0.390	0.257	1.000

Abbreviations: As, arsenic; Hg, mercury; Cd, cadmium; Mn, manganese; Cu, copper; Zn, zinc.

Values are Spearman correlation coefficients. The largest absolute correlation coefficient was 0.390 between Mn and Zn. The maximum adjusted variance inflation factor in the extended linear models was 1.17, indicating no substantial multicollinearity.

Supplemental Table S8. The contextual details of included epidemiological studies

ID	Study	study design	Location	Period	Population (n)	Age(years)	Gender (% male)	Exposure	Detect methods	LOD (μg/L)	Concentrations (mean±SD, ng/ml)	Research Results
1	Liang et al., 2020[1]	Cohort	China	2013-2014	2089	At birth	50.9	Arsenic	ICP-MS	0.0047	2.27 ± 1.51	TSH, FT4
2	Meeker et al., 2009[2]	Cross-sectional	USA	-	219	18-55	100	Arsenic	ICP-MS	4.0	7.97 ± 3.11	TSH
3	Tang et al., 2025[3]	Case–control	China	2016	60	30.7 ± 4.2	0	Arsenic	ICP-MS	-	3.76	Hypothyroidism
4	Zhang et al., 2020[4]	Case–control	China	2017-2019	616	44.7 ± 12.7	22.4	Arsenic Mercury	GF-AAS, ICP-OES	0.03-2.00	Arsenic 135.50 ± 435.35 μg/g Mercury 48.51 ± 57.47 μg/g	Thyroid cancer
5	He et al., 2022[5]	Case–control	China	2017-2019	1170	48.3 ± 13.6	36.4	Arsenic	ICP-OES	-	49.48 ± 61.81	Thyroid cancer
6	Khan et al., 2019[6]	Cross-sectional	Bangladesh	2017	32	16.6 ± 0.7	50	Arsenic	GF-AAS	2	328.36 ± 220.47 μg/g	TSH, T3, FT4, TPOAb
7	Gong et al., 2015[7]	Cross-sectional	USA	-	723	≥40	31.3	Arsenic	ICP-MS	-	5.6 ± 3.0	Hypothyroidism
8	Wu et al., 2025[8]	Cohort	China	-	1351	31.5 ± 3.8	0	Arsenic	ICP-MS	0.3063	17.12 ± 6.33	TSH, T3, T4, FT3, FT4
9	Zhao et al., 2025[9]	Case–control	China	-	236	29.2 ± 4.3	0	Arsenic	ICP-MS, LA-ICP-MS	-	0.02 ± 0.02 μg/g	Hypothyroidism
10	Liu et al., 2021[10]	Case–control	China	2017-2019	394	42.3 ± 10.5	36.6	Mercury	ICP-MS	0.02	1.01 ± 7.32	Thyroid tumor, Thyroid goiter, TSH, FT3, T4
11	Chen et al., 2013[11]	Cross-sectional	USA	2007-2008	Adolescents 1109, Adults 4409	Adolescents 15.5, Adults 46.4	55	Mercury	ICP-MS	0.33	1.62 ± 0.96	TSH, TT3, TT4, FT3, FT4, Tg
12	Rezaei et al., 2019[12]	Cross-sectional	Iran	2018	110	39.4 ± 9.0	40.9	Mercury	ICP-MS	-	0.53 ± 0.3 μg/g	Hypothyroidism
13	Rezaei et al., 2019[12]	Cross-sectional	Iran	2018	110	39.4 ± 9.0	40.9	Mercury	ICP-MS	-	0.41 ± 0.13 μg/g	Hyperthyroidism
14	Rezaei et al., 2019[12]	Cross-sectional	Iran	2018	110	39.4 ± 9.0	40.9	Mercury	ICP-MS	-	0.84 ± 1.1 μg/g	Thyroid cancer
15	Nascimento et al., 2018[13]	Cohort	Brazil	2016	54	10.5 ± 3.2	48.1	Mercury	ICP-MS	-	1.50 ± 0.45	TSH, T3, FT4, TPOAb
16	Shaked et al., 2024[14]	Cross-sectional	USA	2007-2012	7688	>20	52.9	Mercury	ICP-MS	-	17.36 ± 313.5	TPOAb, TgAb

ID	Study	study design	Location	Period	Population (n)	Age(years)	Gender (% male)	Exposure	Detect methods	LOD (μg/L)	Concentrations (mean±SD, ng/ml)	Research Results
17	Kim et al., 2022[15]	Cohort	Korea	2003-2016	5213	49.8±13.1	38.2	Mercury	TDA-AAS	0.01-0.3	1.87± 1.98 μg/g	Thyroid cancer
18	Gallagher et al., 2012[16]	Cross-sectional	USA	2007-2008	2047	52	0	Mercury	-	-	1.44 ± 1.73	TSH, TPOAb, TgAb
19	Llop et al., 2015[17]	Cohort	Spain	2003-2008	1407	30.5±4.2	0	Mercury	AAS	2	7.82 ± 1.60	TSH, TT3, FT4
20	Correia et al., 2020[18]	Cross-sectional	Brazil	2016-2017	110	54.3±6.7	100	Mercury	AAS	0.4589	2.2 ± 2.75	TSH, T3, FT3, FT4, TPOAb, TgAb
21	McGregor & Mason, 1991[19]	Cross-sectional	UK	-	103	39.9±13.2	100	Mercury	-	-	22.1 ± 7.96	TSH
22	Barregård et al., 1994[20]	Cross-sectional	Sweden	-	82	36	100	Mercury	CV-AAS	-	9.446 ± 8.488	TSH, FT3, FT4, TPOAb
23	Reserchers' study	Cohort	China	2017-2018	800	29.53±3.60	0	Arsenic Mercury	ICP-MS	0.05-0.14	Arsenic 0.80 ± 0.72 μg/L Mercury 0.40 ± 0.25 μg/L	TSH, FT4, TPOAb

Abbreviations: TSH, thyroid-stimulating hormone; FT3,Free Triiodothyronine; FT4, free thyroxine; TPOAb, anti-thyroid peroxidase antibody; TgAb, Thyroglobulin Antibodies; T3,Triiodothyronine; T4,Tetraiodothyronine; TT3,Total Triiodothyronine; ICP-MS, Inductively Coupled Plasma Mass Spectrometry; GF-AAS, Graphite Furnace Atomic Absorption Spectrometry; ICP-OES, Inductively Coupled Plasma Optical Emission Spectrometry; LA-ICP-MS, Laser Ablation Inductively Coupled Plasma Mass Spectrometry; TDA-AAS, Thermal Decomposition Amalgamation Atomic Absorption Spectrometry; CV-AAS, Cold Vapor Atomic Absorption Spectrometry.

Supplemental Table S9. The contextual details of included *in vivo* studies

ID	Study	Location	study design	Species	Exposure	Type of model	Mode of exposure	Dose (mg/kg/d)
1	Mukhi et al., 2024[21]	India	Animal Experiment	Rats	$HgCl_2$, As_2O_3	Rats	Oral exposure	Mercury 1.5 arsenic 4
2	Fan et al., 2024[22]	China	Animal Experiment	Rats	$NaAsO_2$	Rats	Oral exposure	2.14
3	Ahmed et al., 2019[23]	Egypt	Animal Experiment	Rats	As_2O_3	Rats	Transplacental exposure	5
4	Xiang et al., 2025[24]	China	Animal Experiment	Rats	$NaAsO_2$	Rats	Oral exposure	8.57
5	Ahangarpour et al., 2017[25]	Iran	Animal Experiment	Mice	$NaAsO_2$	Mice	Oral exposure	50 mg/L
6	Li et al.., 2024[26]	China	Animal Experiment	Rats	$NaAsO_2$	Rats	Oral exposure	10
7	Sun et al.., 2017[27]	China	Animal Experiment	Mice	$NaAsO_2$, Na_2HAsO_4	Mice	Oral exposure	10 μg/L
8	Liu et al, 2006[28]	USA	Animal Experiment	Zebrafish	Na_2HAsO_4	Zebrafish	Others	1 mg/L
9	Biswas et al., 2000[29]	India	Animal Experiment	Goats	$NaAsO_2$	Goats	Oral exposure	25
10	Li et al., 2025[30]	China	Animal Experiment	Zebrafish	Na_2HAsO_4	Zebrafish	Others	50 μg/L
11	Nandheeswari et al., 2024[31]	India	Animal Experiment	Mice	$NaAsO_2$	Mice	Oral exposure	200 μg/L
12	Mohanta et al., 2014[32]	India	Animal Experiment	Cavy	$NaAsO_2$, As_2O_3	Cavy	Oral exposure	50
13	Fan et al., 2023[33]	China	Animal Experiment	Rats	$NaAsO_2$	Rats	Oral exposure	2.5
14	Khan et al, 2019[34]	Pakistan	Animal Experiment	Rabbits	$HgCl_2$	Rabbits	Oral exposure	1.2
15	Zhong et al., 2023[35]	China	Animal Experiment	Zebrafish	$HgCl_2$	Zebrafish	Others	0.1 μg/L
16	Li et al, 2014[36]	China	Animal Experiment	Gobiocypris rarus	$HgCl_2$	Gobiocypris rarus	Others	100μg/L
17	Sun et al, 2018[37]	China	Animal Experiment	Zebrafish	$HgCl_2$	Zebrafish	Others	16 μg/L
18	Kirubagaran et al., 1989[38]	India	Animal Experiment	Clarias batrachus	$HgCl_2$, CH_3HgCl	Clarias batrachus	Others	0.05 mg/L

Supplemental Table S10. The contextual details of included *in vitro* studies

ID	Study	Location	Study design	Exposure Period	cell type	cell source	Exposure	Exposure dose (μM)	Research Results	Dose(μM)
1	Fan et al., 2023[33]	China	Cell Experiments	24,48h	Nthy-ori-3-1	SIBCB(Shanghai Institute of Cell Biology)	$NaAsO_2$	0.1, 1, 10, 50, 100	Cell viability,Morphology, LDH Release,Flow Cytometry	10
2	Trocchianesi et al., 2023[39]	Italy	Cell Experiments	72h	TT	ATCC(American Type Culture Collection)	As_2O_3	10	Cell viability,Cell death	10
3	Fröhlich et al., 2008[40]	Germany	Cell Experiments	24-48h	FTC-238,CGTH-W-1, ONCO-DG-1	ECACC, DSMZ	As_2O_3	0.5-8	Cell viability,Cell death	2
4	Maggisano et al., 2020[41]	Italy	Cell Experiments	24h, 9-18d	Nthy-ori-3-1	Human non-tumorigenic thyroid	MeHg	0.1, 0.5, 1, 2.5, 5	Cell viability,Cell proliferation,Cell cycle,ROS level,Phosphorylation of ERK and AKT	0.1
5	Costa et al., 2007[42]	USA	Cell Experiments	24h	TT	ATCC(American Type Culture Collection)	MeHg	0.5-20	Cell viability,Cell proliferation,IC50	2.3

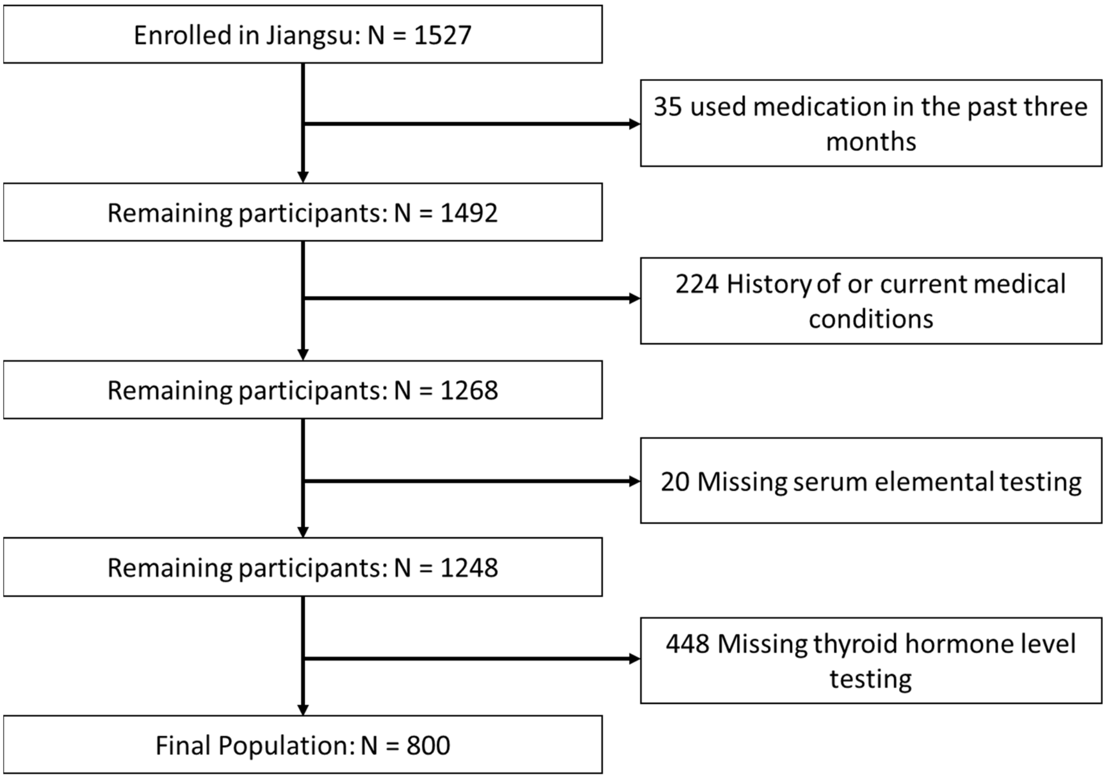

Supplemental Figure S1. Flowchart of the participants selection in the study.

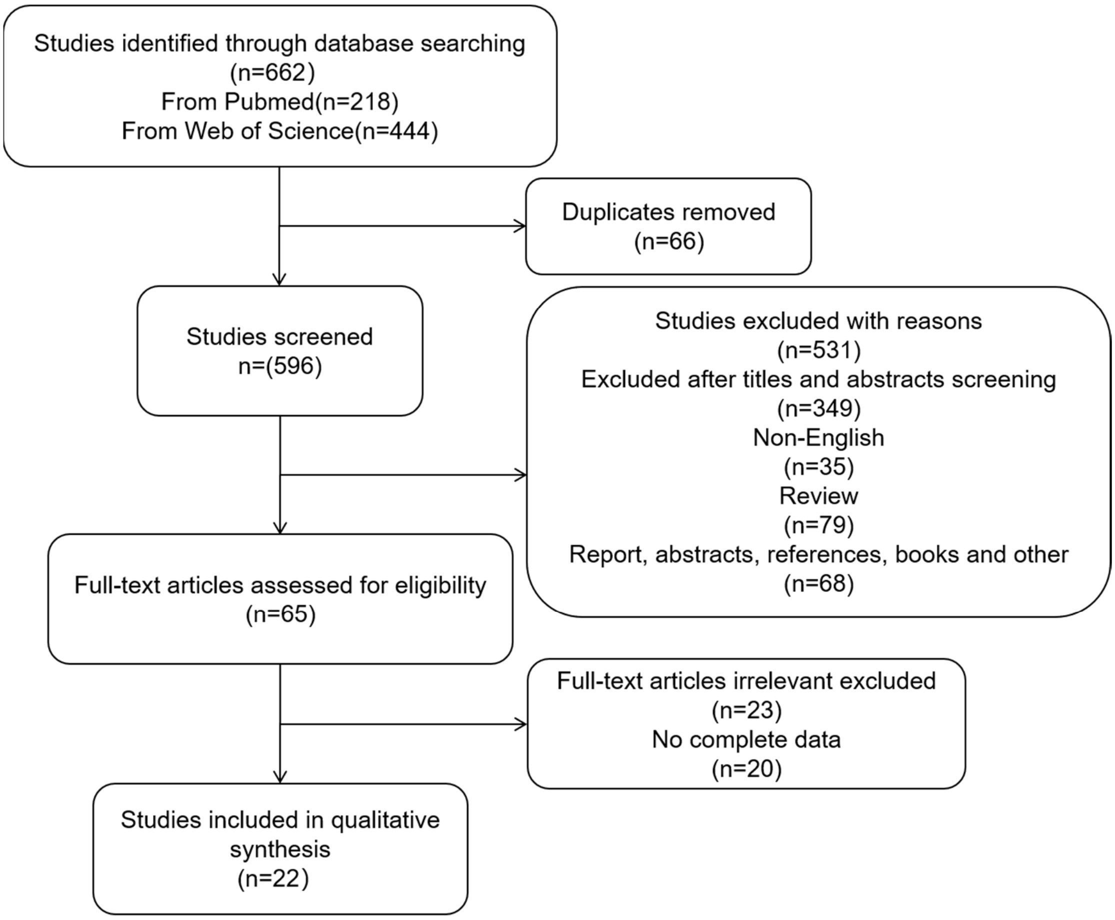

Supplemental Figure S2. **Flow diagram of selecting articles for meta-analysis.**

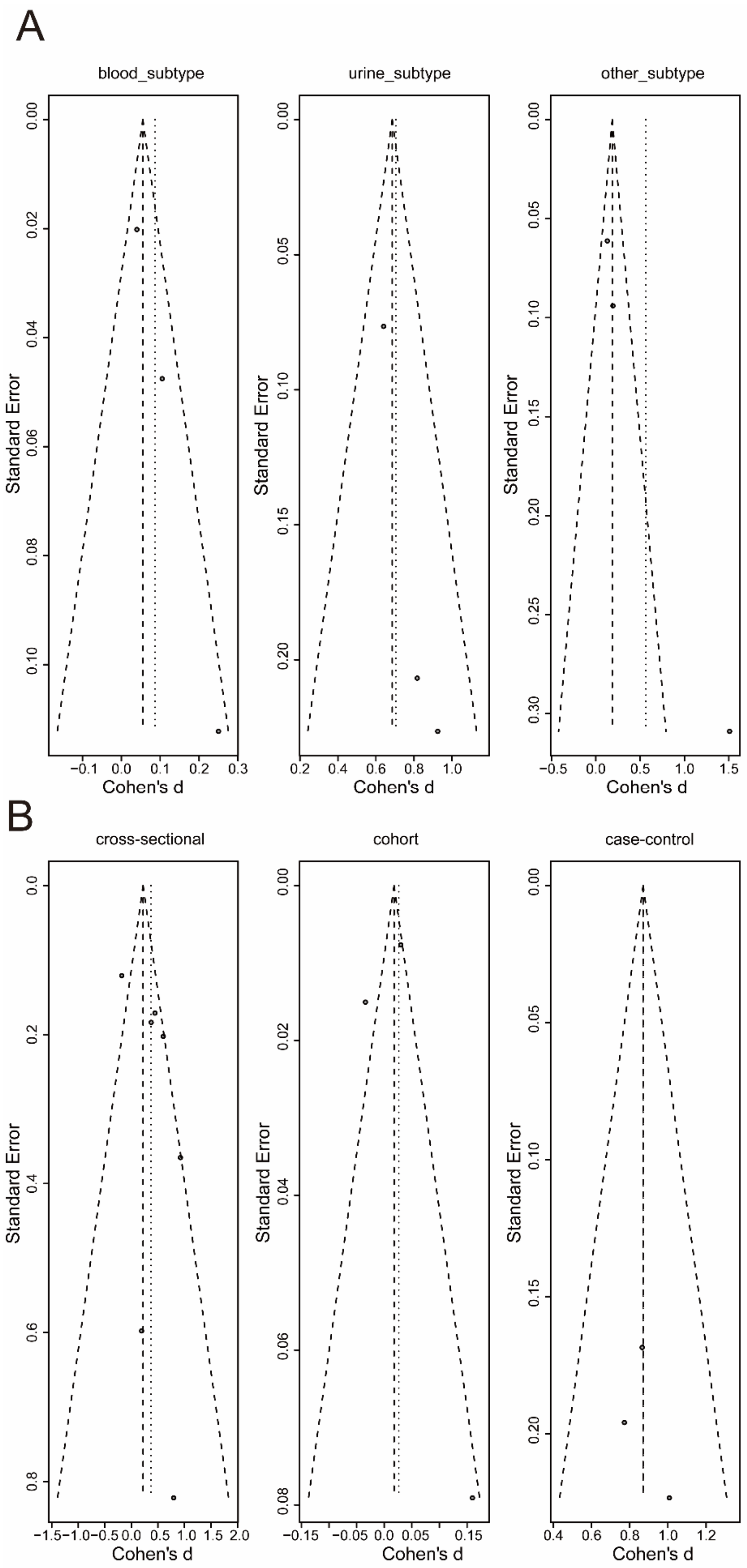

Supplemental Figure S3. Funnel plots for publication bias assessment in meta-analyses on heavy metals and thyroid disorders. (A) Subgroup analysis by biospecimen type for As; (B) Subgroup analysis by study design for Hg. Abbreviations: As, arsenic; Hg, mercury.

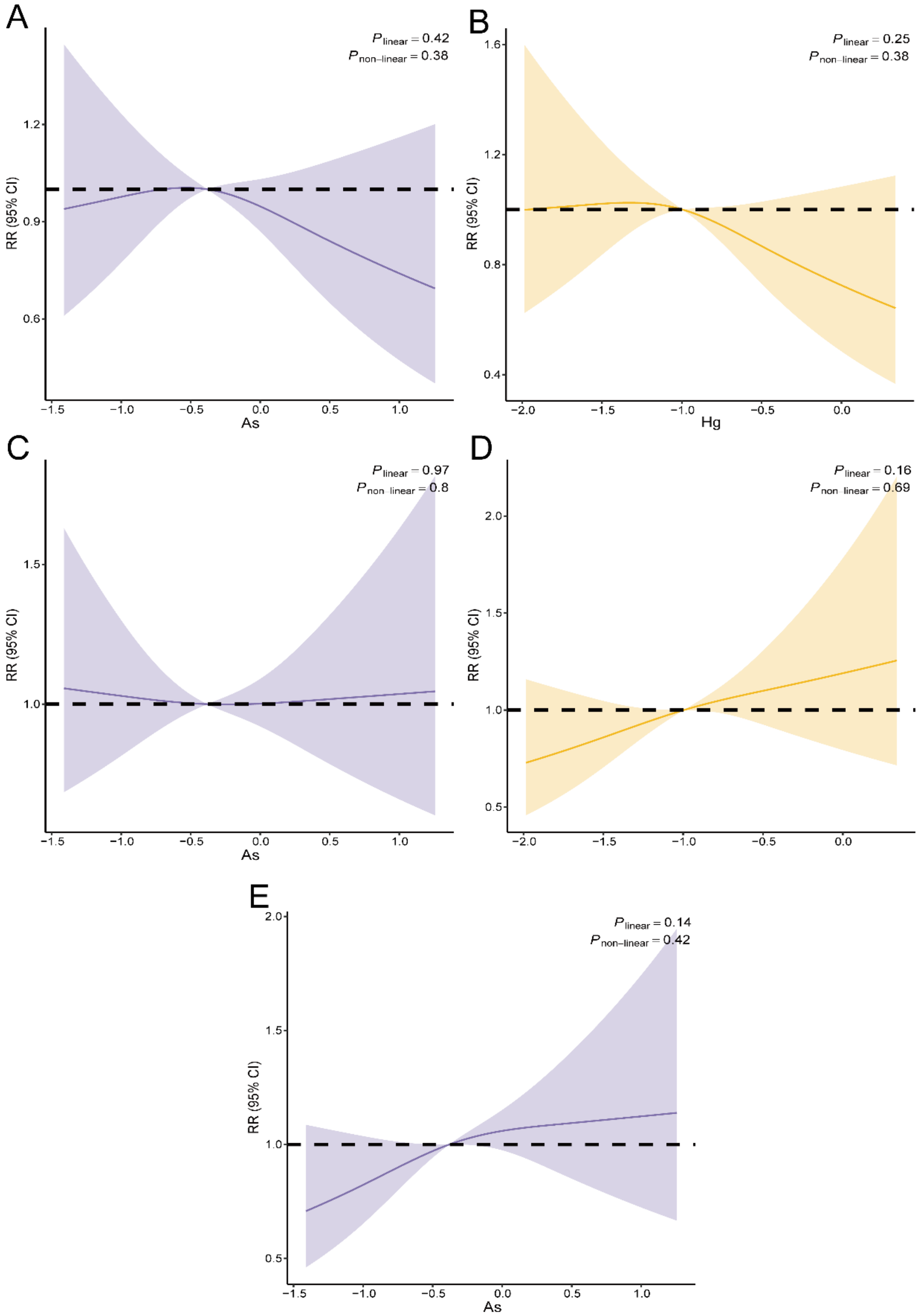

Supplemental Figure S4. Dose–response relationship between As and Hg exposure and thyroid hormone levels. (A) Dose–response relationship between As and TSH in the cohort; (B) Dose–response relationship between Hg and TSH in the cohort; (C) Dose–response relationship between As and TPOAb in the cohort; (D) Dose–response relationship between Hg and TPOAb in the cohort; (E) Dose–response relationship between As and FT4 in the cohort. Abbreviations: RR, relative risk; 95% CI, 95% confidence intervals; As, arsenic; Hg, mercury; FT4, free thyroxine;TSH, thyroid-stimulating hormone; TPOAb, anti-thyroid peroxidase antibody.

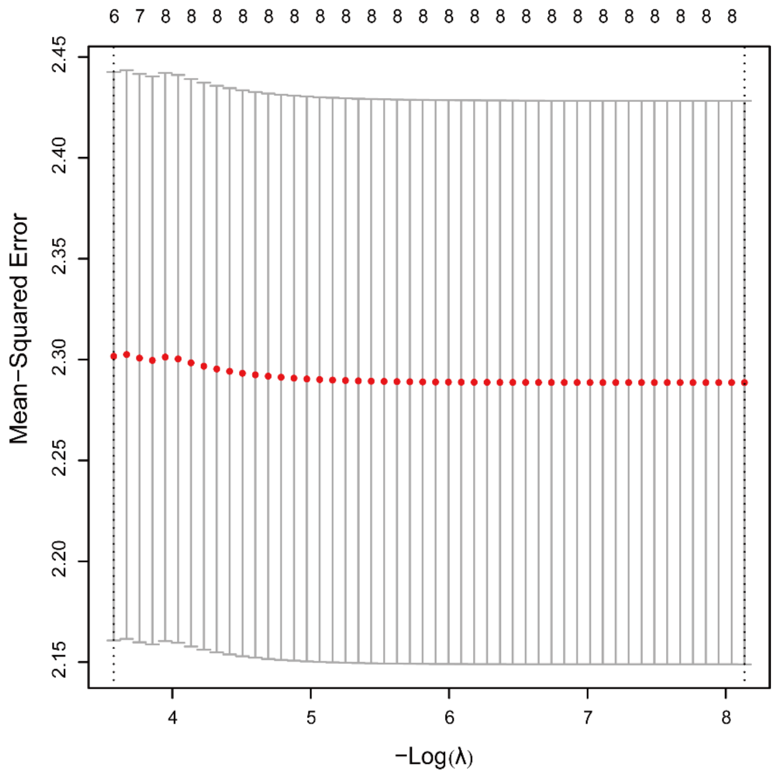

Supplemental Figure S5. Cross-validation curve of FT4-related variables in the cohort. The right vertical dotted line indicates the value of -log(λ) corresponding to the regularization parameter λ that minimizes the cross-validation error.

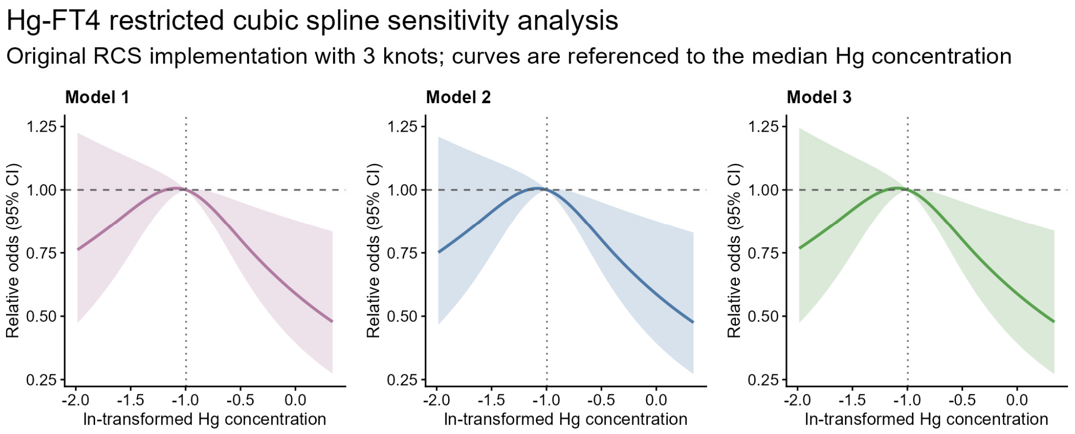

Supplemental Figure S6. Hg–FT4 restricted cubic spline sensitivity analysis after additional covariate adjustment. Model 1 represents the primary covariate-adjusted model; Model 2 additionally adjusts for smoking status; Model 3 further adjusts for Cd, Mn, Cu, and Zn. All models use three knots, the same natural log-transformed Hg scale, and the same median Hg concentration as the reference. Shaded areas represent 95% confidence intervals. Exact *P* values are presented in Supplemental Table S6.

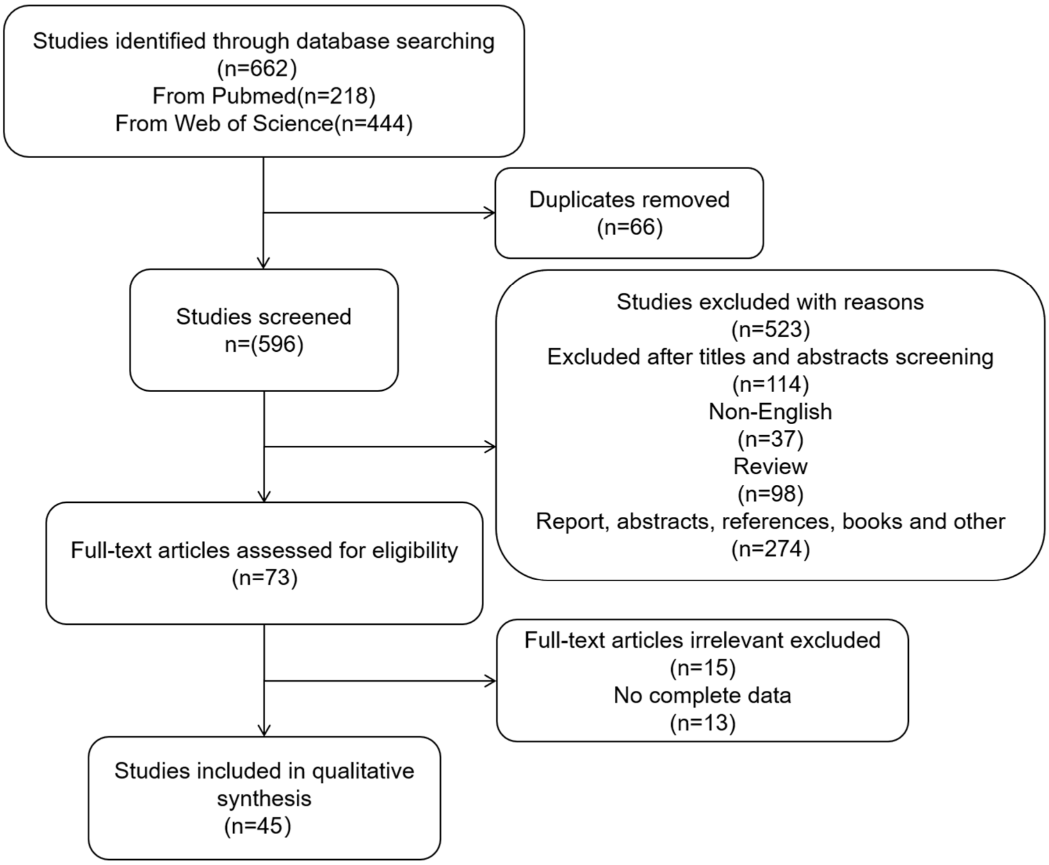

Supplemental Figure S7. Flow diagram of selecting articles for TRAEC-based risk assessment.

Methods and materials supplementation

Study population

The study population was drawn from the cohort based on the study conducted at Nanjing Maternity and Child Health Care Hospital from 2017 to 2018. Baseline serum samples were collected for elemental analysis, with additional follow-up samples collected for thyroid hormone measurement.

A total of 1527 adult women were initially enrolled. Participants from the cohort were excluded according to the following criteria: (i) use of medications affecting thyroid function within the past 3 months (n = 35); (ii) history of or current thyroid dysfunction, hyperlipidemia, or hypertension (n = 224); (iii) lack of serum trace element testing (n = 20); and (iv) lack of serum thyroid hormone level testing (n = 448). After exclusions, 800 adult women were included in the final analysis.

The study protocols involving human participants were approved by the Medical Ethics Committee of Nanjing Maternity and Child Health Care Hospital (003-2017). Informed consent was obtained from all participants. All samples were collected using trace-element-free tubes and stored at -80°C until analysis to prevent contamination and degradation.

Sensitivity analyses.

Sensitivity analyses were conducted to evaluate the robustness of the associations of As and Hg exposure with FT4. The primary model (Model 1) was adjusted for age, BMI, number of pregnancies, diabetes status, occupational type, education level, and exercise frequency, consistent with the primary analysis. Model 2 additionally included smoking status, and Model 3 further included serum Cd, Mn, Cu, and Zn concentrations. All models were based on complete-case analysis, with 800 participants included.

The robustness of the nonlinear association between Hg and FT4 was further examined using restricted cubic spline models with the same knot specification and reference value as in the primary analysis. Model 1 included the same covariates as the primary analysis. Model 2 additionally adjusted for smoking status, whereas Model 3 further adjusted for Cd, Mn, Cu, and Zn.

All additional covariates were complete for the 800 participants included in the analyses. Smoking status was entered as a categorical variable.

References

1. Liang, C.; Han, Y.; Ma, L.; Wu, X.; Huang, K.; Yan, S.; Li, Z.; Xia, X.; Pan, W.; Sheng, J.; et al. Low levels of arsenic exposure during pregnancy and maternal and neonatal thyroid hormone parameters: The determinants for these associations. Environ Int 2020, 145, 106114, doi:10.1016/j.envint.2020.106114.
2. Meeker, J.D.; Rossano, M.G.; Protas, B.; Diamond, M.P.; Puscheck, E.; Daly, D.; Paneth, N.; Wirth, J.J. Multiple metals predict prolactin and thyrotropin (TSH) levels in men. Environ Res 2009, 109, 869-873, doi:10.1016/j.envres.2009.06.004.
3. Tang, C.; Zhou, Y.; He, Y.; Zhang, W.; Huang, Y.; Du, W.; Guo, J.; Chen, G. Association of metal mixtures during the second trimester with gestational hypothyroidism and its mediation by metabolites. Environ Res 2025, 276, 121534, doi:10.1016/j.envres.2025.121534.
4. Zhang, Q.; Hu, M.; Wu, H.; Niu, Q.; Lu, X.; He, J.; Huang, F. Plasma polybrominated diphenyl ethers, urinary heavy metals and the risk of thyroid cancer: A case-control study in China. Environ Pollut 2021, 269, 116162, doi:10.1016/j.envpol.2020.116162.
5. He, J.L.; Wu, H.B.; Hu, W.L.; Liu, J.J.; Zhang, Q.; Xiao, W.; Hu, M.J.; Wu, M.; Huang, F. Exposure to multiple trace elements and thyroid cancer risk in Chinese adults: A case-control study. Int J Hyg Environ Health 2022, 246, 114049, doi:10.1016/j.ijheh.2022.114049.
6. Khan, K.M.; Parvez, F.; Zoeller, R.T.; Hocevar, B.A.; Kamendulis, L.M.; Rohlman, D.; Eunus, M.; Graziano, J. Thyroid hormones and neurobehavioral functions among adolescents chronically exposed to groundwater with geogenic arsenic in Bangladesh. Sci Total Environ 2019, 678, 278-287, doi:10.1016/j.scitotenv.2019.04.426.
7. Gong, G.; Basom, J.; Mattevada, S.; Onger, F. Association of hypothyroidism with low-level arsenic exposure in rural West Texas. Environ Res 2015, 138, 154-160, doi:10.1016/j.envres.2015.02.001.
8. Wu, T.; Li, T.; Zhang, C.; Hao, Y.H.; Zhang, S.W.; Wang, X.P.; Meng, Y.C.; Wu, Y.T.; Huang, H.F. Effects of Maternal Trace Elements Exposure in Early Pregnancy on Maternal Thyroid Hormones and Birth Outcomes. Biol Trace Elem Res 2026, 204, 484-494, doi:10.1007/s12011-025-04691-1.
9. Zhao, Y.; Wang, M.; Chan, Y.-N.; Yang, S.; Wang, P.; Zhou, Y.; Zhang, L.; Wang, H.; Shi, H.; Lum, J.T.-S.; et al. Time-Varying Relationship between Maternal Trace Element Exposure and Gestational Hypothyroidism. Environ. Sci. Technol. Lett. 2025, 12, 390-396, doi:10.1021/acs.estlett.5c00066.
10. Liu, M.; Song, J.; Jiang, Y.; Liu, Y.; Peng, J.; Liang, H.; Wang, C.; Jiang, J.; Liu, X.; Wei, W.; et al. A case-control study on the association of mineral elements exposure and thyroid tumor and goiter. Ecotoxicol Environ Saf 2021, 208, 111615, doi:10.1016/j.ecoenv.2020.111615.
11. Chen, A.; Kim, S.S.; Chung, E.; Dietrich, K.N. Thyroid hormones in relation to lead, mercury, and cadmium exposure in the National Health and Nutrition Examination Survey, 2007-2008. Environ Health Perspect 2013, 121, 181-186, doi:10.1289/ehp.1205239.
12. Rezaei, M.; Javadmoosavi, S.Y.; Mansouri, B.; Azadi, N.A.; Mehrpour, O.; Nakhaee, S. Thyroid dysfunction: how concentration of toxic and essential elements contribute to risk of hypothyroidism, hyperthyroidism, and thyroid cancer. Environ Sci Pollut Res Int 2019, 26, 35787-35796, doi:10.1007/s11356-019-06632-7.
13. Nascimento, S.; Göethel, G.; Gauer, B.; Sauer, E.; Nardi, J.; Cestonaro, L.; Correia, D.; Peruzzi, C.; Mota, L.; Machry, R.V.; et al. Exposure to environment chemicals and its possible role in endocrine disruption of children from a rural area. Environ Res 2018, 167, 488-498, doi:10.1016/j.envres.2018.07.039.
14. Shaked, Y.; Yang, J.; Monaghan, M.; van Gerwen, M. The Association between Metals and Thyroid Cancer in Puerto Rico-A National Health and Nutrition Examination Survey Analysis and Ecological Study. Toxics 2024, 12, doi:10.3390/toxics12090632.
15. Kim, S.; Song, S.H.; Lee, C.W.; Kwon, J.T.; Park, E.Y.; Oh, J.K.; Kim, H.J.; Park, E.; Kim, B. Low-Level Environmental Mercury Exposure and Thyroid Cancer Risk Among Residents Living Near National

Industrial Complexes in South Korea: A Population-Based Cohort Study. Thyroid 2022, 32, 1118-1128, doi:10.1089/thy.2022.0084.

16. Gallagher, C.M.; Meliker, J.R. Mercury and thyroid autoantibodies in U.S. women, NHANES 2007-2008. Environ Int 2012, 40, 39-43, doi:10.1016/j.envint.2011.11.014.
17. Llop, S.; Lopez-Espinosa, M.J.; Murcia, M.; Alvarez-Pedrerol, M.; Vioque, J.; Aguinagalde, X.; Julvez, J.; Aurrekoetxea, J.J.; Espada, M.; Santa-Marina, L.; et al. Synergism between exposure to mercury and use of iodine supplements on thyroid hormones in pregnant women. Environ Res 2015, 138, 298-305, doi:10.1016/j.envres.2015.02.026.
18. Correia, M.M.; Chammas, M.C.; Zavariz, J.D.; Arata, A.; Martins, L.C.; Marui, S.; Pereira, L.A.A. Evaluation of the effects of chronic occupational exposure to metallic mercury on the thyroid parenchyma and hormonal function. Int Arch Occup Environ Health 2020, 93, 491-502, doi:10.1007/s00420-019-01499-0.
19. McGregor, A.J.; Mason, H.J. Occupational mercury vapour exposure and testicular, pituitary and thyroid endocrine function. Hum Exp Toxicol 1991, 10, 199-203, doi:10.1177/096032719101000309.
20. Barregård, L.; Lindstedt, G.; Schütz, A.; Sällsten, G. Endocrine function in mercury exposed chloralkali workers. Occup Environ Med 1994, 51, 536-540, doi:10.1136/oem.51.8.536.
21. Mukhi, S.; Manjrekar, P.A.; Srikantiah, R.M.; Harish, S.; Kotian, H.; Rao, Y.L.; Sherly, A. Evaluation of the cognitive, physiological, and biomarker effects of heavy metal exposure in Wistar rats. Vet World 2024, 17, 1855-1863, doi:10.14202/vetworld.2024.1855-1863.
22. Fan, L.; Song, Q.; Jin, Y.; He, R.; Diao, H.; Luo, P.; Wang, D. Prolonged exposure to NaAsO(2) induces thyroid dysfunction and inflammatory injury in Sprague–Dawley rats, involvement of NLRP3 inflammasome–mediated pyroptosis. Arch Toxicol 2024, 98, 3673-3687, doi:10.1007/s00204-024-03837-9.
23. Ahmed, R.G.; El-Gareib, A.W. Gestational Arsenic Trioxide Exposure Acts as a Developing Neuroendocrine-Disruptor by Downregulating Nrf2/PPARγ and Upregulating Caspase-3/NF-κB/Cox2/BAX/iNOS/ROS. Dose Response 2019, 17, 1559325819858266, doi:10.1177/1559325819858266.
24. Xiang, J.; Fan, L.; Li, H.; Song, Q.; Jin, Y.; He, R.; Pan, X.; Wang, D. Molecular disturbances and thyroid gland dysfunction in rats chronically exposed to a high dose of $NaAsO_2$: Insights from proteomic and phosphoproteomic analyses. J Hazard Mater 2025, 484, 136746, doi:10.1016/j.jhazmat.2024.136746.
25. Ahangarpour, A.; Alboghobeish, S.; Oroojan, A.A.; Zeidooni, L.; Samimi, A.; Afshari, G. Effects of Combined Exposure to Chronic High-Fat Diet and Arsenic on Thyroid Function and Lipid Profile in Male Mouse. Biol Trace Elem Res 2018, 182, 37-48, doi:10.1007/s12011-017-1068-1.
26. Li, H.; Xiang, J.; Song, Q.; Jin, Y.; Zhou, M.; Fan, L.; Wang, D. Active Vitamin D Ameliorates Arsenite-Induced Thyroid Dysfunction in Sprague-Dawley Rats by Inhibiting the Toll-like Receptor 4/NF-KappaB-Mediated Inflammatory Response. Toxics 2024, 12, doi:10.3390/toxics12120887.
27. Sun, H.J.; Li, S.W.; Li, C.; Wang, W.Q.; Li, H.B.; Ma, L.Q. Thyrotoxicity of arsenate and arsenite on juvenile mice at organism, subcellular, and gene levels under low exposure. Chemosphere 2017, 186, 580-587, doi:10.1016/j.chemosphere.2017.08.042.
28. Liu, F.J.; Wang, J.S.; Theodorakis, C.W. Thyrotoxicity of sodium arsenate, sodium perchlorate, and their mixture in zebrafish Danio rerio. Environ Sci Technol 2006, 40, 3429-3436, doi:10.1021/es052538g.
29. Biswas, U.; Sarkar, S.; Bhowmik, M.K.; Samanta, A.K.; Biswas, S. Chronic toxicity of arsenic in goats: clinicobiochemical changes, pathomorphology and tissue residues. Small Rumin Res 2000, 38, 229-235, doi:10.1016/s0921-4488(00)00162-0.
30. Li, T.; Zhang, D.; Ding, L.; Zhou, H.; Hou, Y.; Hong, H.; Sun, H.; Yu, X. Transcriptomic Analysis of Endocrine System Responses in Zebrafish Embryos Following Exposure to Environmentally Relevant Concentrations of Arsenate. Fishes 2025, 10, 97, doi:https://doi.org/10.3390/fishes10030097.

31. Nandheeswari, K.; Jayapradha, P.; Nalla, S.V.; Dubey, I.; Kushwaha, S. Arsenic-Induced Thyroid Hormonal Alterations and Their Putative Influence on Ovarian Follicles in Balb/c Mice. Biol Trace Elem Res 2024, 202, 4087-4100, doi:10.1007/s12011-023-03988-3.
32. Mohanta, R.K.; Garg, A.K.; Dass, R.S.; Behera, S.K. Blood biochemistry, thyroid hormones, and oxidant/antioxidant status of guinea pigs challenged with sodium arsenite or arsenic trioxide. Biol Trace Elem Res 2014, 160, 238-244, doi:10.1007/s12011-014-0041-5.
33. Fan, L.; He, Z.; Wang, L.; Gaoyang, H.; Wang, D.; Luo, P. Alterations of Bax/Bcl-2 ratio contribute to NaAsO(2) induced thyrotoxicity in human thyroid follicular epithelial cells and SD rats. Ecotoxicol Environ Saf 2023, 264, 115449, doi:10.1016/j.ecoenv.2023.115449.
34. Khan, R.; Ali, S.; Mumtaz, S.; Andleeb, S.; Ulhaq, M.; Tahir, H.M.; Khan, M.K.A.; Khan, M.A.; Shakir, H.A. Toxicological effects of toxic metals (cadmium and mercury) on blood and the thyroid gland and pharmacological intervention by vitamin C in rabbits. Environ Sci Pollut Res Int 2019, 26, 16727-16741, doi:10.1007/s11356-019-04886-9.
35. Zhong, L.; Zhang, H.; Wu, L.; Ru, H.; Wei, N.; Yao, F.; Ni, Z.; Duan, X.; Li, Y. Thyroid-Disrupting Effects of Cadmium and Mercury in Zebrafish Embryos/Larvae. Water 2023, 15, 135, doi:https://doi.org/10.3390/w15010135.
36. Li, Z.H.; Chen, L.; Wu, Y.H.; Li, P.; Li, Y.F.; Ni, Z.H. Alteration of thyroid hormone levels and related gene expression in Chinese rare minnow larvae exposed to mercury chloride. Environ Toxicol Pharmacol 2014, 38, 325-331, doi:10.1016/j.etap.2014.07.002.
37. Sun, Y.; Li, Y.; Liu, Z.; Chen, Q. Environmentally relevant concentrations of mercury exposure alter thyroid hormone levels and gene expression in the hypothalamic-pituitary-thyroid axis of zebrafish larvae. Fish Physiol Biochem 2018, 44, 1175-1183, doi:10.1007/s10695-018-0504-2.
38. Kirubagaran, R.; Joy, K.P. Toxic effects of mercurials on thyroid function of the catfish, Clarias batrachus (L.). Ecotoxicol Environ Saf 1989, 17, 265-271, doi:10.1016/0147-6513(89)90046-8.
39. Trocchianesi, S.; Po, A.; Citarella, A.; Spinello, Z.; Rughetti, A.; Besharat, Z.M.; Autilio, T.M.; Pecce, V.; Verrienti, A.; Elisei, R.; et al. Molecular mechanisms of the tyrosine kinase inhibitor pralsetinib activity in in-vitro models of medullary thyroid carcinoma: Aberrant activation of the HH-Gli signaling pathway in acquired resistance. Biomed Pharmacother 2023, 164, 114995, doi:10.1016/j.biopha.2023.114995.
40. Fröhlich, E.; Czarnocka, B.; Brossart, P.; Wahl, R. Antitumor effects of arsenic trioxide in transformed human thyroid cells. Thyroid 2008, 18, 1183-1193, doi:10.1089/thy.2008.0114.
41. Maggisano, V.; Bulotta, S.; Celano, M.; Maiuolo, J.; Lepore, S.M.; Abballe, L.; Iannone, M.; Russo, D. Low Doses of Methylmercury Induce the Proliferation of Thyroid Cells In Vitro Through Modulation of ERK Pathway. Int J Mol Sci 2020, 21, doi:10.3390/ijms21051556.
42. Costa, L.G.; Fattori, V.; Giordano, G.; Vitalone, A. An in vitro approach to assess the toxicity of certain food contaminants: methylmercury and polychlorinated biphenyls. Toxicology 2007, 237, 65-76, doi:10.1016/j.tox.2007.05.003.
